# Perfect association between spatial swarm segregation and the X-chromosome speciation island in hybridizing *Anopheles coluzzii* and *Anopheles gambiae* populations

**DOI:** 10.1038/s41598-022-14865-9

**Published:** 2022-06-24

**Authors:** Abdoulaye Niang, Hamidou Maïga, Simon P. Sawadogo, Lassana Konaté, Ousmane Faye, Yoosook Lee, Roch K. Dabiré, Abdoulaye Diabaté, Frederic Tripet

**Affiliations:** 1 Institut de Recherche en Sciences de la Santé, Bobo-Dioulasso, Burkina Faso; 2 Centre for Applied Entomology and Parasitology, School of Life Sciences, Keele University, Staffordshire, UK; 3 Laboratoire d’Ecologie Vectorielle et Parasitaire, Faculté des Sciences et Techniques, Université Cheikh Anta Diop, Dakar, Sénégal; 4 Florida Medical Entomology Laboratory, Department of Entomology and Nematology, Institute of Food and Agricultural Sciences, University of Florida, Vero Beach, FL 32962 USA

**Keywords:** Molecular evolution, Behavioural genetics

## Abstract

The sibling species *An. coluzzii* and *An. gambiae* s.s. are major malaria vectors thought to be undergoing sympatric speciation with gene flow. In the absence of intrinsic post-zygotic isolation between the two taxa, speciation is thought possible through the association of assortative mating and genomic regions protected from gene flow by recombination suppression. Such genomic islands of speciation have been described in pericentromeric regions of the X, 2L and 3L chromosomes. Spatial swarm segregation plays a major role in assortative mating between sympatric populations of the two species and, given their importance for speciation, genes responsible for such pre-mating reproductive barriers are expected to be protected within divergence islands. In this study 2063 male and 266 female *An. coluzzii* and *An. gambiae* s.s. individuals from natural swarms in Burkina Faso, West Africa were sampled. These were genotyped at 16 speciation island SNPs, and characterized as non-hybrid individuals, F_1_ hybrids or recombinant F_1+n_ backcrossed individuals. Their genotypes at each speciation island were associated with their participation in *An. coluzzii* and *An. gambiae*-like swarms. Despite extensive introgression between the two species, the X-island genotype of non-hybrid individuals (37.6%), F_1_ hybrids (0.1%) and F_1+n_ recombinants (62.3%) of either sex perfectly associated to each swarm type. Associations between swarm type and the 3L and 2L speciation islands were weakened or broken down by introgression. The functional demonstration of a close association between spatial segregation behaviour and the X speciation island lends further support to sympatric speciation models facilitated by pericentric recombination suppression in this important species complex.

## Introduction

Speciation is a fundamental biological process that involves the evolution of reproductive isolation between diverging populations. Whilst the general processes of geographical isolation, genetic divergence and evolution of post-mating reproductive isolation characterising allopatric speciation are better known^1^, those involved in sympatric speciation, the emergence and divergence of two gene pools within a panmictic population, are still poorly understood^2,3^. Theoretical models have shown that this requires genes of local adaptations to become associated with those of mate choice^4–7^. In the face of ongoing gene flow, this association is thought to occur only under a restricted set of genetic and environmental conditions. Under some circumstances, strong divergent natural selection might negate the effect of gene flow at very small spatial scales and could be the primary driver of speciation^1,8^. Features of genomes such as chromosomal inversions, peri-centromeric regions and other regions characterized by reduced recombination can also facilitate the association of local adaptation and assortative mating loci^2,5–7^. In addition, sex chromosomes are thought to promote the rapid accumulation of pre- and post-mating isolation genes due to their hemizygosity and lower recombination rate^9^. Currently, teasing out the genomic signature of the onset of speciation from the genomic processes that follow the establishment of intrinsic post-mating reproductive isolation constitutes a major challenge and there are few natural model systems that allow it^2,10,11^.

The *Anopheles gambiae* complex comprises a number of important vectors of human malaria in Africa that are separated by various degrees of reproductive isolation^12,13^. Amongst its different cryptic taxa, two sibling species, *An. coluzzii* and *An. gambiae* s.s., formerly known as M and S molecular forms^14^ are undergoing speciation with gene flow and may provide the ideal model system for studying the genomic signature of pre-mating isolation independent of intrinsic post-mating isolation speciation processes^15^. The two sibling species interbreed freely in the laboratory and do not exhibit intrinsic post-mating barriers to reproduction in the form of hybrid inviability or sterility^16,17^. In studies based on natural sympatric populations from Central and Eastern West Africa, hybrids were typically found to be uncommon or rare^18–20^. A longitudinal study conducted over a period of two decades in Mali shows that the reproductive isolation between M and S is unstable. A strong assortative mating that sustained the maintenance of the two populations, is periodically disrupted by episodes of hybridization, indicating temporal variation in hybridization rates^21^ due to the decreases of reproductive isolation followed by selections against hybrids. However, surveys undertaken in coastal areas West Africa in Senegal and The Gambiae have uncovered hybridization zones where hybrids frequencies between *An. coluzzii* and *An. gambiae* s.s. as high as 24% can be observed^22–24^.

In the absence of intrinsic post-mating reproductive barriers between the sibling species, their genetic identity is thought to be maintained through the combined effects of strong assortative mating, as evidenced by studies conducted in Mali and Burkina Faso^18,25^, and through extrinsic post-mating barriers to reproduction in the form of decreased hybrid fitness^26^. Whilst the later has yet to be demonstrated experimentally, evidence of ecological divergence between the two sibling species has accumulated, suggesting that hybrids could incur fitness costs in a variety of ways. The two sub-taxa have been shown to differ in preferred larval breeding sites^27,28^ and ability to detect and avoid aquatic predators. Entomological surveys and laboratory experiments have also shown that Sub-Saharan^29–31^ populations of *An. coluzzii* exhibit higher tolerance to desiccation stress and dominate vector populations in drier seasons and habitats^32–34^. In the same regions, the sibling species also differ in their aestivation strategies^35,36^ affecting their survival and/or migration strategy during the dry season. However, despite these differences, the sibling species co-exist in vast areas of West Africa and are characterized by similar levels of anthropophily and endophily.

Both sibling species mate in mating aggregations called swarms that are initiated at dusk usually within villages^37^. However, they tend to differ in the height at which they form swarms and the type of ground markers they use^37^. Such swarm spatial segregation has been observed in Burkina Faso and Mali and may play a major role in assortative mating between the sibling species^38–40^. However, mixed swarms are sometimes found at low frequencies^41–44^ suggesting that short-range mate recognition mechanisms must also be involved in assortative mating^40,45^. One such mechanism could be flight-tones harmonic convergence^46,47^ whilst the possible role of contact pheromones remains to be adequately explored^40,48^.

That hybridization results in gene flow between the two sibling species in spite of strong assortative mating has been supported by genome-wide population genetic studies first based on Short Tandem Repeat (STRs) markers^49,50^ and next on Single Nucleotide Polymorphisms (SNPs). The former revealed a mosaic pattern of genetic differentiation with most of the genome lacking differentiation because of ongoing gene flow and limited areas of the genome seemingly protected from recombination^49,50^; the later identified those regions more precisely through their higher marker density^51,52^. These revealed three highly genetically-differentiated regions located near the centromeres of chromosomes X, 2L and 3L suggesting that sympatric speciation in these incipient species probably involved the divergence of such ‘islands of speciation' possibly containing clusters of speciation genes and protected from gene flow through recombination suppression^51,52^. As of today, these three regions are the only regions that have been detected by high-density genome scans across the whole sympatric range of the two sibling species^21,24,53,54^. That current hybridization plays a role in the speciation process is further supported by recent studies that used Divergent Island SNPs (DIS) genotyping to distinguish heterozygous F_1_ hybrids from the recombinant genotypes of F_1+n_ backcrosses in sympatric populations^55^. Studies based on DIS confirmed the occurrence of varying levels of hybridization across sympatric populations of the sibling species translating into varying degrees of introgression^56^. As an example, a recent introgression event occurred in Burkina Faso and Mali in which the entire 2L speciation island passed from *An. gambiae* s.s. into *An. coluzzii* through hybridization and selection resulting in the transfer of important pesticide resistance loci between the two species^53,55,57^. Interestingly, the DIS data also suggests that these populations are now regaining their specific X, 2L and 3L pericentric regions, highlighting the fact that selection does act against recombinant genotypes. Selection against F_1+n_ backcrosses would also explain how pericentromeric islands of speciation remain significantly associated in the hybrid zones of Western coastal West Africa despite high levels of gene flow^22–24^.

Whist the extent of genetic differentiation in other areas of the genome and how much of it is due to selection for local adaptation is currently debated, the X, 2L and 3L speciation islands seem to have played and still appear to be playing a crucial role in the genomic structure of speciation in these species. The close association between the X speciation island and pre-mating isolation genes was recently experimentally demonstrated by swapping the X-island of *An. coluzzii* with that of *An. gambiae* s.s. through multigenerational selective introgression^15^. Females from the resulting recombinant strains differing only at their X-chromosome island strictly mated with males which had matching island type^15^. Because assortative mating was female driven and occurred in small cages, it highlighted a short-range mechanism, possibly involving male-expressed specific cues and female choice, and independent of the process of swarm spatial segregation.

Given the importance of spatial swarm segregation for assortative mating in natural sympatric populations of the sibling species, one would expect genes for swarming site preference to be also associated with one of the pericentromeric islands of divergence. In this study, for the first time, this association was formally tested using thousands of males and females from *An. coluzzii* and *An. gambiae* s.s. directly collected from natural swarms in sympatric populations from Burkina Faso. DIS^56^ was then used to genotype all individuals. Thus, in contrast to what is commonly done, and to avoid potential bias due to circularity, *An. coluzzii* and *An. gambiae* swarms were described using individual DIS genotypes rather than rely solely on one species diagnostic based on a X-linked locus^44^. Thereafter, the association between the genotypes of non-hybrid individuals and 1st generation (F_1_) and 1 + *n*th generation hybrid males and females (F_1+n_) at the X, 2L and 3L islands were associated with swarm spatial segregation. The results demonstrate the close association between the X-island and spatial swarm segregation initiated by males. In addition, there was a strong but significantly weaker association with the 3L island. The association between swarm type and the 2L island was broken down by recent adaptive introgression from *An. gambiae* s.s. to *An. coluzzii*. These results lend further support to an island-of-speciation mode of sympatric speciation in which core assortative mating and divergent ecological adaptation genes are genetically linked and protected from gene flow through pericentric recombination suppression.

## Results

### DIS genotyping and genotypic composition of swarms

A total of 2063 males from a total of 106 swarms was collected in Bana, Soumousso and VK7. They were genotyped for DIS and characterized as ‘pure’ *An. coluzzii* and *An. gambiae*, and F_1_ and F_1+n_ hybrids (Table 1). F_1_ hybrid males were very rare, with only 2 found over the course of the study (0.1%), whilst F_1+n_ hybrids were extremely abundant and made for 62.3% (N = 1285) of individuals. Hybrids and backcrosses were assigned to *An. coluzzii* and *An. gambiae* based on the majority of their DIS alleles—i.e. *An. coluzzii* or *An. gambiae* dominated the genetic background. Next, the swarms were described as monospecific when composed entirely of *An. coluzzii* and *An. gambiae* males, or mixed if composed of males assigned as both *An. coluzzii* and *An. gambiae*. The resulting swarm genetic structure indicated that, despite often being composed of many recombinant males, 103 (97.2%) swarms were monospecific and only 3 (2.8%) swarms mixed (Table 1).

**Table 1:** Number and percentage of *An. coluzzii* and *An. gambiae*, and F_1_ and F_1+n_ hybrid males from monospecific and mixed swarms.

Sampling location		Swarm characteristics			Parental species				Hybrids			
					*An. coluzzii*		*An. gambiae*		F_1_		F_1+n_	
Locality	Year	Type	N swarms	N Total	N	(%)	N	(%)	N	(%)	N	(%)
Bana	2012	*An. coluzzii*	16	65	1	1.5	0	0	0	0	64	98.5
Bana	2012	*An. gambiae*	9	57	0	0	57	100	0	0	0	0
Soumousso	2006	*An. gambiae*	2	28	0	0	26	92.9	0	0	2	7.1
Soumousso	2007	*An. coluzzii*	2	36	30	83.3	0	0	0	0	6	16.7
Soumousso	2007	*An. gambiae*	13	374	0	0	356	95.2	0	0	18	4.8
Soumousso	2008	*An. gambiae*	12	176	0	0	164	93.2	1	0.6	11	6.3
Soumousso	2008	Mixed	1	8	4	50	1	12.5	0	0	3	37.5
Soumousso	2011	*An. coluzzii*	1	15	1	6.7	0	0	0	0	14	93.3
Soumousso	2011	Mixed	1	8	1	12.5	3	37.5	0	0	4	50
Soumousso	2011	*An. gambiae*	7	74	0	0	73	98.6	0	0	1	1.4
Soumousso	All	*An. coluzzii*	3	51	31	60.8	0	0	0	0	20	39.2
Soumousso	All	*An. gambiae*	34	652	0	0	619	94.9	1	0.2	32	4.9
Soumousso	All	Mixed	2	16	5	31.3	4	25	0	0	7	43.8
Soumousso	All	All	39	719	36	5	623	86.6	1	0.1	59	8.2
VK7	2006	*An. coluzzii*	2	67	17	25.4	0	0	0	0	50	74.6
VK7	2006	*An. gambiae*	1	16	0	0	15	93.8	0	0	1	6.3
VK7	2006	Mixed	1	24	8	33.3	1	4.2	0	0	15	62.5
VK7	2008	*An. coluzzii*	24	493	9	1.8	0	0	0	0	484	98.2
VK7	2011	*An. coluzzii*	14	622	9	1.4	0	0	1	0.2	612	98.4
VK7	All	*An. coluzzii*	40	1182	35	3	0	0	1	0.1	1146	97
VK7	All	*An. gambiae*	1	16	0	0	15	93.8	0	0	1	6.3
VK7	All	Mixed	1	24	8	33.3	1	4.2	0	0	15	62.5
VK7	All	All	42	1222	43	3.5	16	1.3	1	0.1	1162	95.1
Total	All	*An. coluzzii*	59	1298	67	5.1	0	0	1	0.1	1230	94.8
Total	All	*An. gambiae*	44	725	0	0	691	95.3	1	0.1	33	4.6
Total	All	Mixed	3	40	13	32.5	5	12.5	0	0	22	55
Grand Total	All	All	106	2063	80	3.9	696	33.7	2	0.1	1285	62.3

Swarms were collected from Bana (2012), Soumousso (2006, 2007, 2008 and 2011) and VK7 (2006, 2008 and 2011). The swarm type (monospecific *An. coluzzii* and *An. gambiae* s.s. or mixed with males of both species), number of swarms sampled, and total number of individuals genotyped are indicated. All means the total number of swarms per type or per village sampled during the period of the study.

### Hybrid frequencies in the sibling species

**Figure 1:**
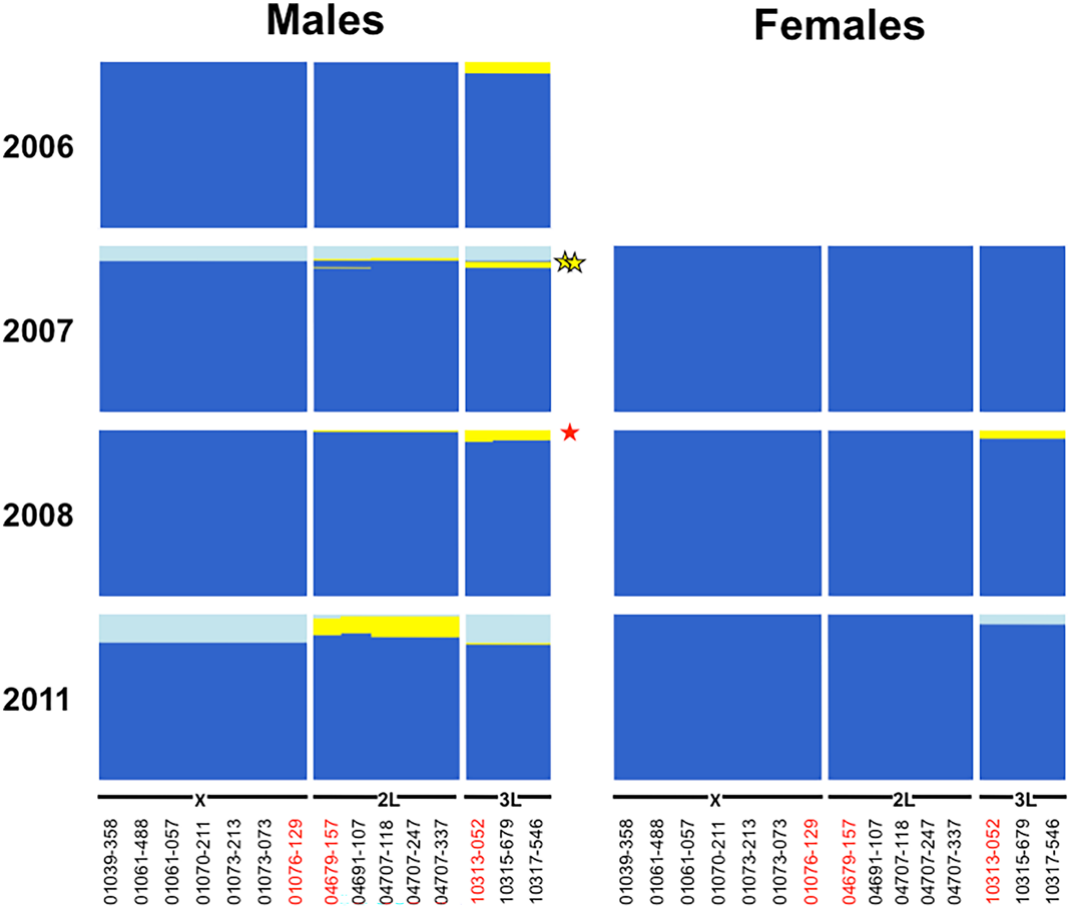
DIS map of males and females *Anopheles coluzzii* and *An. gambiae* collected from swarms in the village of Somousso in Burkina Faso—DIS genotypes, specific to *An. coluzzii* (light blue) and *An. gambiae* (dark blue) are shown; herozygous DIS genotypes are in yellow—SNPs nearest to centromeres are in red lettering. One F_1_ hybrid male with *An. gambiae* maternal X-island was identified in 2008 (red star) and 2 males captured in 2007 were characterized by an *An. coluzzii* X-islands but *An. gambiae* 2L and 3L islands (yellow stars).

**Figure 2:**
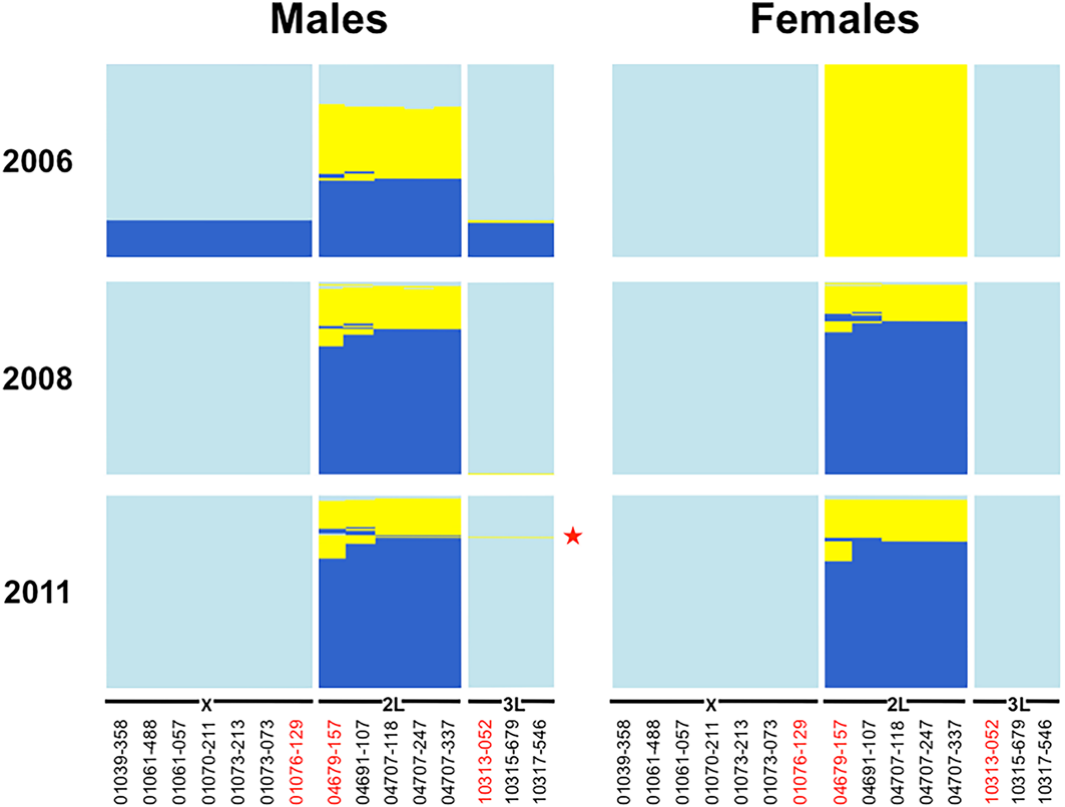
DIS map of males and females *Anopheles coluzzii* and *An. gambiae* collected from swarms in the village of VK7 in Burkina Faso—DIS genotypes specific to *An. coluzzii* (light blue) and *An. gambiae* (dark blue) are shown; heterozygous DIS genotypes are in yellow—SNPs nearest to centromeres are in red lettering. One F_1_ hybrid male with *An. coluzzii* maternal X-island was identified in 2011 (red star).

Male F_1+n_ hybrids were much more abundant in *An. coluzzii* swarms*,* where they made up 94.8% of individuals. *An. gambiae* s.s., made up only 4.6% and were characterized by recombinant DIS genotypes (Likelihood-ratio Chi-square: *χ*^2^ = 1878.3, df = 1, *n* = 2021, *P* < 0.001). The frequency of *An. coluzzii* recombinant genotypes significantly increased over the course of the study in VK7 and Soumousso but this increase was significantly sharper in Soumousso where the starting frequency of recombinant was only 16.7% in 2007 but reached 93.3% in 2011 (Logistic regression: *n* = 1228, Location: *χ*^2^ = 0.001, df = 1, *P* = ns; Year: *χ*^2^ = 57.4, df = 1, *P* < 0.001; interaction: *χ*^2^ = 4.9, df = 1, *P* = 0.027) (Table 1). The two F_1_ hybrid males differed in the ancestry of their maternal X chromosome alleles. One was captured in 2008 in Soumousso and resulted from an *An. gambiae* female cross-mating with an *An. coluzzi* male (Fig. 1). The other hybrid male was caught in VK7 in 2011 and resulted from an *An. coluzzii* female mating with an *An. gambiae* male (Fig. 2).

Amongst the 266 females collected from mating pairs, similar patterns of hybridizations were observed than in males, albeit no F_1_ hybrid were captured (Table 2). F_1+n_ backcrossed genotypes were very common (72.9%). They made up 99% of all females *An. coluzzii* but only 4.4% of *An. gambiae* s.s. females (Likelihood-ratio Chi-square: *χ*^2^ = 257.5, df = 1, *n* = 260, *P* < 0.001). Unfortunately, no females were captured from the comparatively rare *An. coluzzii* swarms from Soumousso.

**Table 2:** Number and percentage of *An. coluzzii* and *An. gambiae*, and F_1_ and F_1+n_ hybrid females from monospecific and mixed swarms.

Sampling location		Swarm characteristics			Parental species				Hybrids			
					*An. coluzzii*		*An. gambiae*		F1		F1 + n	
Locality	Year	Type	N swarms	N total	N	(%)	N	(%)	N	(%)	N	(%)
Soumousso	2007	*An. coluzzii*	0	0	0	0	0	0	0	0	0	0
Soumousso	2007	*An. gambiae*	5	14	0	0	14	100	0	0	0	0
Soumousso	2008	*An. gambiae*	11	39	0	0	37	94.9	0	0	2	5.1
Soumousso	2008	Mixed	1	1	0	0	1	100	0	0	0	0
Soumousso	2011	*An. coluzzii*	0	0	0	0	0	0	0	0	0	0
Soumousso	2011	Mixed	1	3	0	0	3	100	0	0	0	0
Soumousso	2011	*An. gambiae*	7	15	0	0	14	93.3	0	0	1	6.7
Soumousso	All	*An. coluzzii*	0	0	0	0	0	0	0	0	0	0
Soumousso	All	*An. gambiae*	23	68	0	0	65	95.6	0	0	3	4.4
Soumousso	All	Mixed	2	4	0	0	4	100	0	0	0	0
Soumousso	All	All	25	72	0	0	69	95.8	0	0	3	4.2
VK7	2006	*An. coluzzii*	1	1	0	0	0	0	0	0	1	100
VK7	2006	*An. gambiae*	0	0	0	0	0	0	0	0	0	0
VK7	2006	Mixed	1	1	0	0	1	100	0	0	0	0
VK7	2008	*An. coluzzii*	27	142	1	0.7	0	0	0	0	141	99.3
VK7	2011	*An. coluzzii*	12	50	1	2	0	0	0	0	49	98
VK7	All	*An. coluzzii*	40	193	2	1	0	0	0	0	191	99
VK7	All	*An. gambiae*	0	0	0	0	0	0	0	0	0	0
VK7	All	Mixed	1	1	0	0	1	100	0	0	0	0
VK7	All	All	41	194	2	1	1	0.5	0	0	191	98.4
Total	All	*An. coluzzii*	40	193	2	1	0	0	0	0	191	99
Total	All	*An. gambiae*	23	68	0	0	65	95.6	0	0	3	4.4
Total	All	Mixed	3	5	0	0	5	100	0	0	0	0
Grand Total	All	All	66	266	2	0.8	70	26.3	0	0	194	72.9

Females were collected from swarms in Soumousso (2006, 2007, 2008 and 2011) and VK7 (2006, 2008 and 2011). The swarm type (monospecific *An. coluzzii* and *An. gambiae* s.s. or mixed with males of both species), number of swarms sampled, and total number of individuals genotyped are indicated. All means the total number of swarms per type or per village sampled during the period of the study.

### Recombinant genotypes and islands of divergence

The majority of *An. coluzzii* and *An. gambiae* male recombinants (83.7 and 93.9% respectively) and female recombinants (72.8 and 100%) captured in swarms had DIS genotypes compatible with the introgression of one or several entire islands (heterozygous at all SNPs within a chromosome). The remainder of individuals had more complex genotypes with evidence of recombination within heterospecific introgressed islands (mixture of homozygous and heterozygous loci within 1 or several islands) (Figs. 1, 2, Supplementary Tables S1–S2). In *An. coluzzii* males and females*,* backcrossed hybrid genotypes at islands of divergence mostly occurred through introgression of the 2L island, with only a single male recombinant for the 3L pericentric region. In *An. gambiae*, only 4 males and no females were recombinant on 2L, but 29 males and 3 females were recombinant for 3L (Figs. 1, 2, Supplementary Tables S1–S2). Importantly, recombination amongst loci within the X chromosome pericentric island was never observed. Interestingly, 2 (0.1%) males captured in Soumousso in 2007 were characterized by an *An. coluzzii* X-islands but not *An. gambiae* 2L and 3L islands. According to our allelic majority rule, these males were therefore considered as *An. gambiae* backcrosses (Supplementary Tables S1–S2). No females heterozygous for the X-island were found.

### Association between swarm type and divergence islands

**Figure 3:**
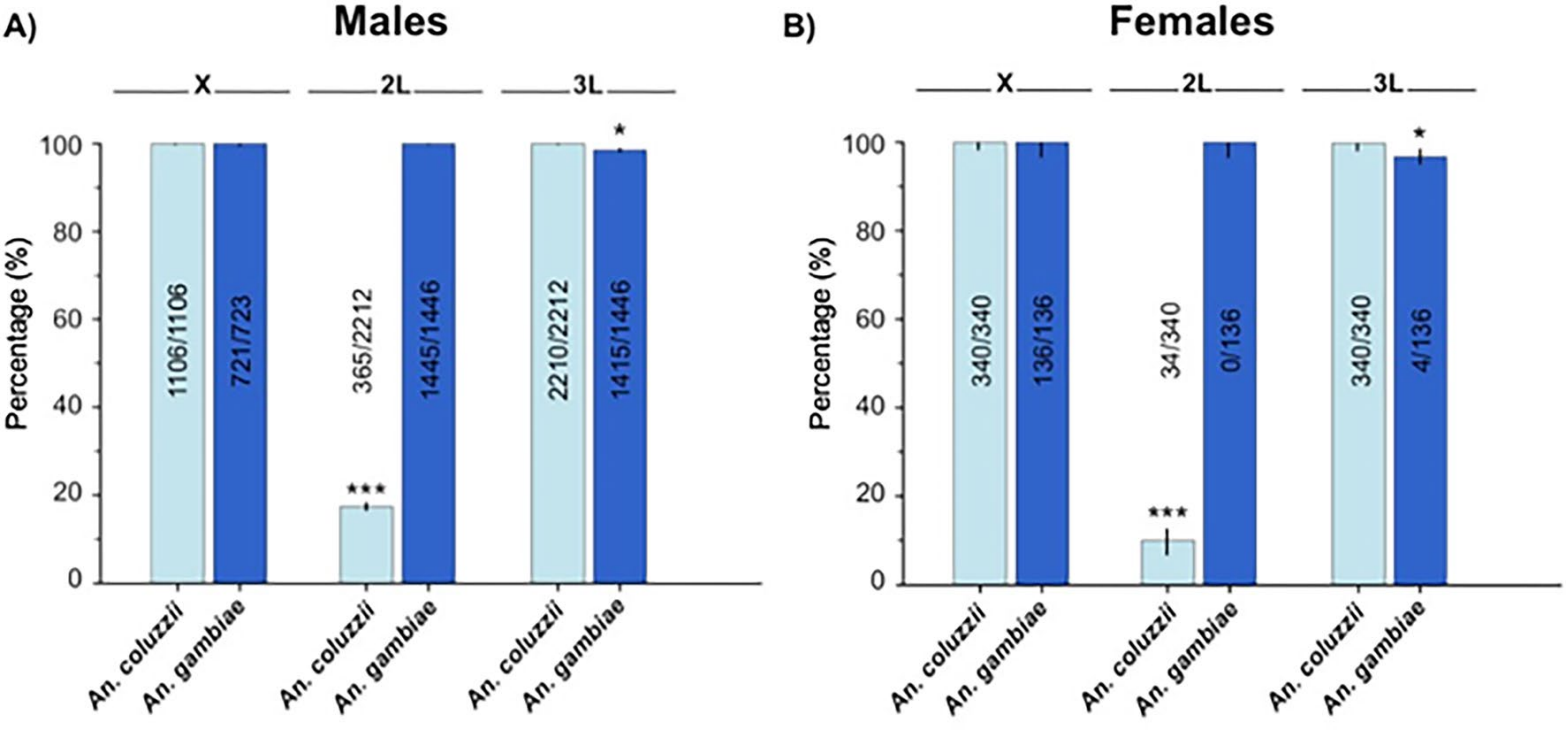
Association between swarm type and divergence islands—Relative proportions of pericentric islands genotypes matching that of the swarm they were captured from for each chromosome in males (**A**) and females (**B**). Sample sizes are indicated and vertical whiskers show 95% confidence intervals. *P*-values are: *P* < 0.001***, *P* < 0.01**, *P* < 0.05*.

An examination was made of the association between swarm spatial segregation and the X, 2L and 3L speciation islands, first in males who initiate swarms, and then in females (Fig. 3). The relative proportions of pericentric islands genotypes matching that of the swarm they were captured from were compared among chromosomes by Logistic Regressions. The analyses were conducted for each sex, first on homozygous non-recombinants individuals and then recombinant individuals characterized by introgression of one or several entire heterospecific islands (Fig. 3, Table 3). Next, examination included individuals with evidence of recombination within heterospecific islands which had no bearing on the results giving the low numbers of such genotypes.

**Table 3:** Logistic regressions testing the effect of chromosome on the strength of association between pericentromeric island genotypes and swarm type.

Recombinants	Swarm	Sex	*n*	Likelihood ratio	*P*-value	Pairwise comparisons LR *P*-values^a^		
						X vs 2L	X vs 3L	2L vs 3L
Entire island(s) only	*An. coluzzii*	Males	5530	5034.2	< 0.001	< 0.001	0.203	< 0.001
–	*An. coluzzii*	Females	1020	1025.1	< 0.001	< 0.001	1.000	< 0.001
–	*An. gambiae*	Males	3615	41.32	< 0.001	0.239	< 0.001	< 0.001
–	*An. gambiae*	Females	408	8.87	0.012	1.000	0.018	0.018
All recombinants^b^	*An. coluzzii*	Males	6490	5984.1	< 0.001	< 0.001	0.203	< 0.001
–	*An. coluzzii*	Females	1158	1167.5	< 0.001	< 0.001	1.000	< 0.001
–	*An. gambiae*	Males	3625	41.32	< 0.001	0.239	< 0.001	< 0.001
–	*An. gambiae*	Females	408	8.87	0.012	1.000	0.018	0.018

^a^Recombinant chromosomes included those characterized by one or two entire heterospecific divergence islands, and recombinants with parts of or recombined heterospecific island(s) leading to identical results.

^b^The *P*-values of likelihood-ratio (LR) tests on odds ratios generated by the logistic regression. All pairwise comparisons were also tested using Fisher–Irwin tests and *N *− 1 Chi-square tests robust to low cell counts yielding comparable results (see “Methods”).

The analyses revealed that *An. coluzzii* and *An. gambiae* male and females strictly associated with swarms that matched their X island genotype. This simple rule was the simplest and most parsimonious explanation for the distribution of island genotypes among monospecific swarms. It accounted for the spatial swarm distribution of 100% of *An. coluzzii* males and females, as well as 100% of *An. gambiae* females and 99.7% of *An. gambiae* males (Fig. 3). Further analyses revealed that one of the two males characterized by *An. gambiae* 2L and 3L genotypes had an *An. coluzzii*-like X chromosome (Supplementary Table S1) and, had the rDNA ITS marker locus typical of *An. gambiae.* Because this marker is located closer to the X centromere than the DIS markers, this indicated a rare recombination event within the X-chromosome island. The other male was *An. coluzzii*-like at the rDNA marker and was therefore the only exception to the rule.

Despite the high level of introgression between the sibling species, a near perfect association between the 3L and swarm specificity was found in *An. coluzzii*. The strength of association between genotype and swarm type did not significantly differ between the X and 3L islands in this species (Table 3), suggesting that the X and 3L islands are potentially strongly genetically associated. This contrasted with *An. gambiae,* where introgressed *An. coluzzii* islands characterized 2.1% (31/1446) and 3.0% (4/136) of male and female 3L chromosomes resulting in a significantly weaker association between swarm type and the 3L island (Likelihood Ratio test on odds ratio: *P* = 0.012) than the association with the X island (Fig. 3, Table 3). Finally, high levels of introgression of the 2L divergence island from *An. gambiae* to *An. coluzzii* obliterated any association between 2L and swarm type in the later species. However, in *An. gambiae,* the 2L was as strongly associated with swarm type as the X-island, with only a single male detected heterozygous for the 2L pericentromeric genomic area (Fig. 3, Table 3, Supplementary Table S1).

The DIS genotypic composition of the 3 mixed swarms was also examined to verify that swarm segregation was again associated with the X-chromosome. However, the data from mixed swarms was largely uninformative because all 3 mixed swarms were dominated by *An. coluzzii* males homozygous across all 3 islands, with very few recombinants (Supplementary Table S3). Rare *An. gambiae* males and females captured in these *An. coluzzii* swarms were all homozygous at all DIS loci (Supplementary Table S3).

### Recombinant frequencies at the larval, indoor resting and swarming stages

The hypothesis that male and female recombinants at the divergent island loci might be present at the larval and/or indoor resting adult stages but would not contribute to swarms was tested by comparing their frequency for all three types of collections made in the villages VK7 and Soumousso (Supplementary Tables S4–S7). The overall frequencies of introgressed X, 2L and 3L islands for each chromosome at each life stages, were compared for each species and sex using Logistic Regression (Table 4). In all 4 models, there was a large significant effect on the frequency of individuals with introgressed pericentric islands as described above. However, no significant differences between the larval, swarming adult and resting indoor stages were found suggesting that recombinants at divergence loci were represented equally and that none of the sampled life stages were selected against (Table 4).

**Table 4:** Logistic regressions testing the effect of chromosome and life stage on frequencies of recombinant genotypes.

Species	Sex	Source	*n*	df	LR chi-square	*P*-value
*An. coluzzii*	Males	Chromosome	7665	2	2067.7	< 0.001
		Live stages		2	2.2	0.337
	Females	Chromosome	2568	2	649.8	< 0.001
		Live stages		2	4.7	0.098
*An. gambiae*	Males	Chromosome	5315	2	118.8	< 0.001
		Live stages		2	6.3	0.043
	Females	Chromosome	2424	2	62.2	< 0.001
		Live stages		2	1.6	0.445

The frequencies of introgressed X, 2L and 3L islands (entire islands) were compared among samples from larvae, adult resting indoors and swarming adults. Interactions were tested but not significant.

### Kdr introgression and species-specific 2L island recovery

**Figure 4:**
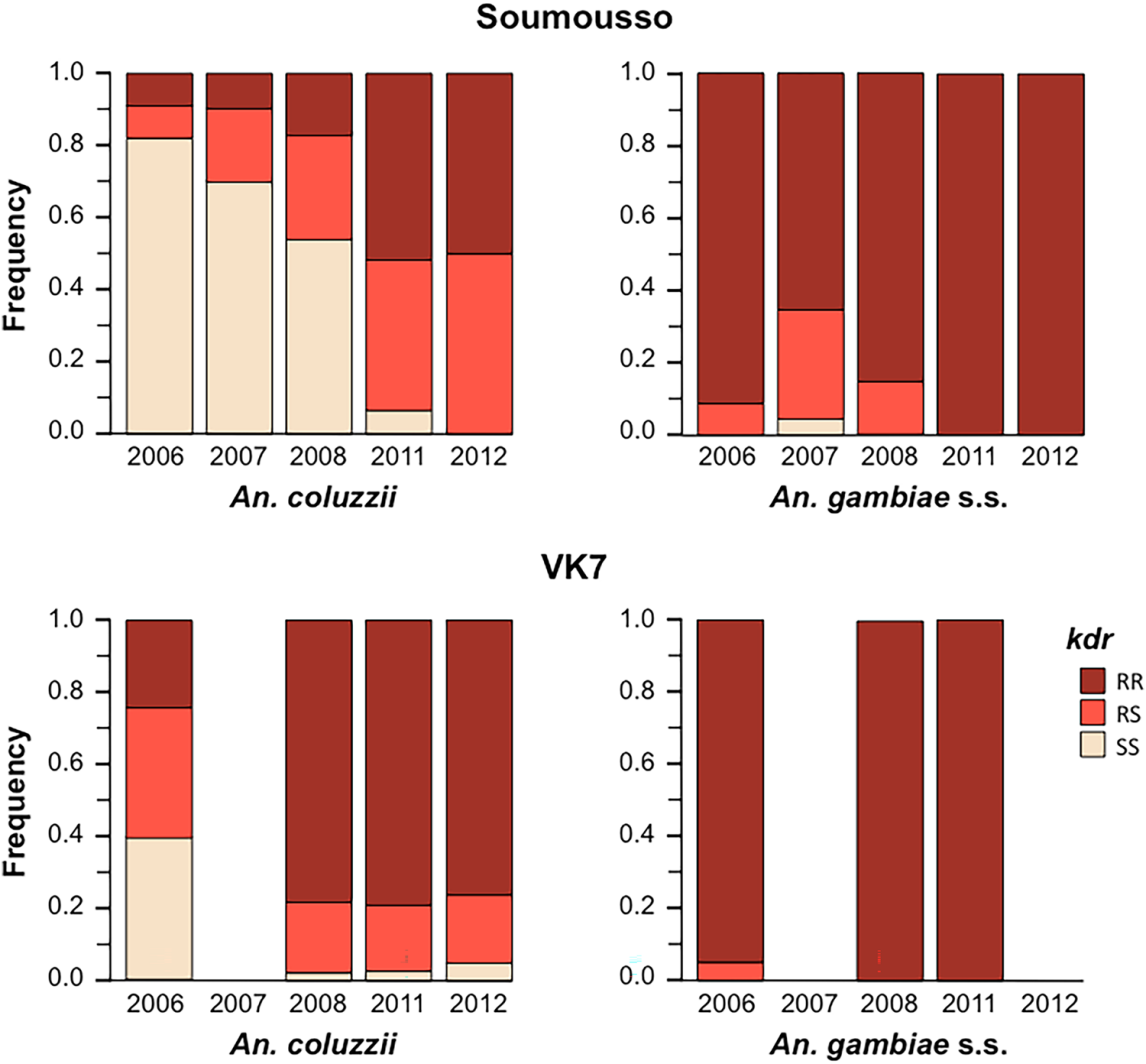
Changes in frequencies of introgressed *kdr* locus—The proportion of *An. coluzzii* individuals carrying the resistant allele (RR: homozygous *kdr* genotype or RS: heterozygous *kdr* genotypes) increased drastically from 2006 to near fixation in 2012 due to selective introgression of the 2L island from *An. gambiae*. The proportion of *An. coluzzii* individuals carrying the susceptible allele (SS: homozygous susceptible genotype) decreased over the same period.

The presence of the knockdown resistance (*kdr*) allele against pyrethroid within the 2L island from *An. gambiae* is thought to have driven its recent adaptive introgression into *An. coluzzii* in areas under extensive bednet coverage^54^. Compatible with this hypothesis, the frequency of homozygous (RR) and heterozygous (RS) *kdr* genotypes in *An. coluzzii* increased sharply from 2006 to 2012 in VK7 and Soumousso (Fig. 4). In contrast, it was found at high frequencies in *An. gambiae* throughout the study (Fig. 4).

**Figure 5:**
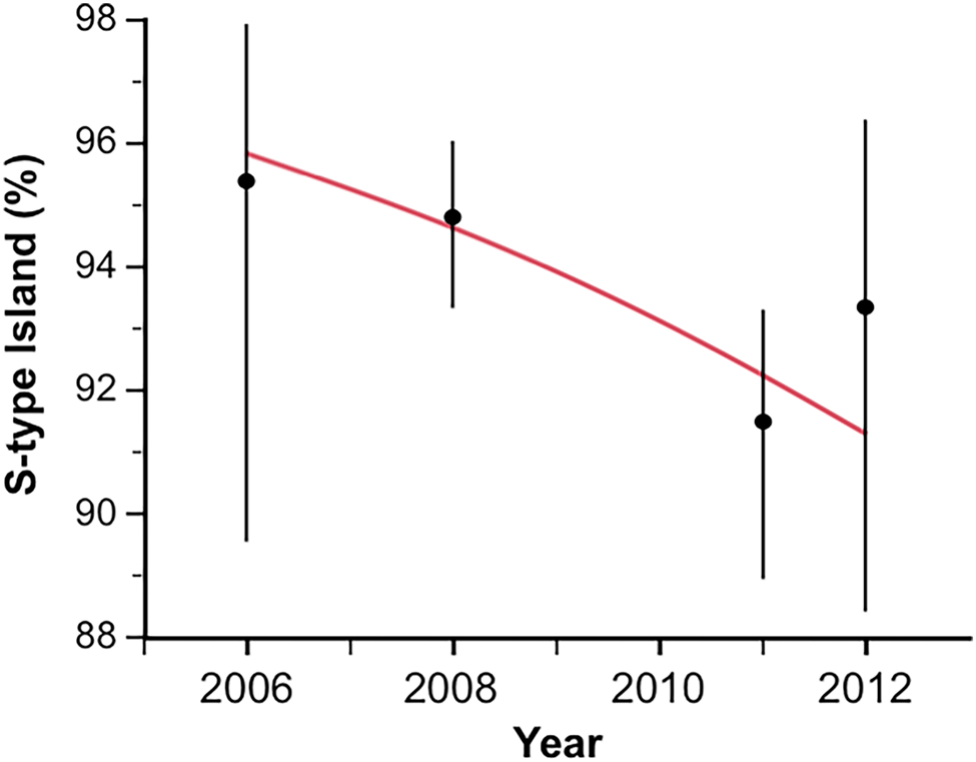
*Kdr* introgression and species-specific 2L island recovery in *An. coluzzii*—Following the initial selective introgression of the 2L *An. gambiae* island into *An. coluzzii* new recombinant 2L-islands haplotypes featuring an *An. coluzzii*-like pericentromeric region and the introgressed *kdr* allele appeared. A new selective sweep associated with these haplotypes quickly led to a decrease in the frequency of *An. gambiae* pericentromeric 2L-island loci in *An. coluzzii.*

Whilst recombination amongst DIS loci within the X island was never observed and was very rare in the 3L island (2 in 6856 chromosomes), the 2L pericentric region recently introgressed into *An. coluzzii* and was broken down by recombination much more commonly (288 in 6856 chromosomes) (Supplementary Table S8). This was also the case in the subset of individuals genotyped at the *kdr* locus (Supplementary Table S9). If *An. coluzzii* recombinants individuals with mixed island genotypes would be selected against, recombinant 2L island featuring an *An. coluzzii*-like loci with the resistant *kdr* allele should have a strong fitness advantage. This hypothesis was tested by comparing the frequency of *An. coluzzii* 2L islands bearing such characteristics in VK7, where samples sizes allowed such comparisons. This analysis confirmed that the *An. coluzzii* population is progressively regaining its specific 2L island after having integrated the resistant *kdr* allele (Likelihood-ratio Chi-square: *n* = 2082, df = 1, *P* = 0.013) (Fig. 5).

## Discussion

Over the last decade, much progress has been made in understanding the behavioural mechanisms responsible for assortative mating between the sibling species of the *An. gambiae* complex^18,40,58^. In particular, an extensive body of work has demonstrated the role of swarm spatial segregation in premating reproductive isolation between *An. coluzzii* and *An. gambiae*^26,44,59^. This study has highlighted for the first time the genetic architecture underlying swarm spatial segregation by using extensive swarm sampling combined with DIS genotyping in Burkina Faso, a region of West Africa with recent hybridization and adaptive introgression between *An. coluzzii* and *gambiae*^21,55^. Compatible with these events, the frequency of F_1+n_ backcrosses genotypes at three known speciation islands^49,52^, increased overtime and were more frequent in *An. coluzzii* indicating imbalanced bidirectional introgression between the two sibling species. Using a simple majority rule, all backcrosses could be assigned to *An. coluzzii* or *An. gambiae* genetic backgrounds allowing for the characterization of *An. coluzzi* and *An. gambiae*-like swarms. Finally, the association between male and female genotypes at the X, 2L and 3L speciation islands and each swarm type was tested, revealing a perfect association between the X-island genotypes and swarm-type and lending further support to an island-of-speciation model of sympatric speciation facilitated by pericentric recombination suppression^15^. The weakened association with the 2L and 3L islands was due to strong introgression of the 2L island of *An. gambiae* into *An. coluzzii* and weak introgression of 3L from *An. coluzzii* to *An. gambiae*.

Overall, the frequencies of recombinant DIS genotypes observed in *An. coluzzii* at VK7 and Soumousso were high and increased from 2006 to 2012 but were comparatively rare in *An. gambiae*. Compared to F_1+n_ recombinants, F_1_ hybrid genotypes were extremely rare, with only 2 F_1_ male (one with a *An. coluzzii*-like maternal X and one with an *An. gambiae* one) no F_1_ female hybrids were found over the course of the study. These results suggest that hybridization is relatively rare whilst introgression is sustained through strong directional selection of the 2L island of speciation once passed on to *An. coluzzii*^21,53,55,56^*.* These patterns mirror those inferred from a DIS genotyping study conducted on samples from nearby Mali spanning 1991 to 2012, which showed low frequencies of hybridization between the sibling species except for rare episodic pulses^21^. Currently, the ecological and genetic factors that may determine the occurrence of such events are poorly known. Recently, Niang et al.^26^ suggested that the extreme asymmetric dominance by *An. coluzzii* over *An. gambiae* observed at certain times of the year in rice-growing areas of Burkina Faso could lead to temporary breakdowns in assortative mating. Thus, hybridization may be occurring regularly but in very narrow set of ecological settings that cannot usually be picked-up by usual mosquito sampling schemes. For example, a study conducted in Mali in 2006 demonstrated that the massive distribution of ITNs during the 2000s leading to a drastic reduction in populations was followed by hybridization episodes characterised by the occurrence of F_1_ hybrids. As a result, *An. coluzzii* acquired the pyrethroid resistance previously known only in *An. gambiae* through adaptive introgression indicated by increased rates of F_1+n_ within the sympatric populations^21^. A hybridization zone was also reported in the extreme west Africa region^22,23,60^.

In this study, even though a large majority of recombinant genotypes identified involved introgression of an entire island, individuals with recombination within islands were also found. In *An. coluzzii,* most recombinant DIS genotypes involved introgression of the entire *An. gambiae* 2L island bearing the *kdr* resistance allele, albeit there were 4.2% of genotypes showing recombination within the 2L islands. In contrast, this was never observed within the X island and was infrequent within the 3L island in recombinants of either species. In *An. coluzzii*, recombination within the 2L island led to the emergence of haplotypes that combine the *kdr* resistant allele with *An. coluzzii* alleles at SNP loci nearest to the centromere^55^. Concurrent with increased insecticide-treated bednets distribution^61^, these haplotypes significantly increased in frequency within the time frame of this study suggesting a strong fitness advantage over other *An. coluzzii* 2L DIS recombinants individuals^55^.

Overall, the distribution and frequencies of DIS recombinant genotypes found in this study are compatible with comparative genomic studies conducted in sympatric populations across West Africa. These have shown no evidence of recombination in the X-island while adaptive introgression was repeatedly observed in autosomal centromeric areas^54,57^. The exception to this rule being the hybrid zone identified in the Western-most part of West Africa and where recombination within the X-island has been documented^62,63^.

Whilst our results show a large significant effect of chromosome on the frequency of individuals with introgressed pericentric islands, no significant difference between the larval, swarming adult and resting indoor stages were found, suggesting that recombinants at divergence loci were represented equally at each of the sampled life stages and therefore that there is no stage-specific selection against recombinant genotypes. Because positively-selected recombinants involving the 2L island dominate our samples, we cannot rule out the possibility that X and 3L recombinants are selected against.

The most important finding of this study is that the X chromosome island genotype was found to perfectly associated to the spatial segregation of swarms of *An. coluzzii* and *An. gambiae*. This was true both for male and female swarming individuals, with the exception of a single male (1 out of 2329) carrying an *An. coluzzii*-like X chromosome in an *An. gambiae* typical swarm. The association between swarm type and the 3L island was significantly weaker than the association with the X-island and completely broken for the 2L island. In *An. coluzzii* the 3L island was strongly associated with swarm type as was the X island, while in *An. gambiae* the strength of association between genotype and swarm type was not significantly different between the 2L and the X islands. These results suggested that the high level of introgression of the 2L divergent island from *An. gambiae* to *An. coluzzii* would prevent any association between the 2L island and the swarm type. Moreover, the X and 3L islands would be strongly genetically associated in *An. coluzzii*. Finally, the weakened association with the 2L and 3L islands confirms directional adaptive introgression of the 2L island of *An. gambiae* into *An. coluzzii* as well as some level of introgression of 3L from *An. coluzzii* to *An. gambiae*. Using a laboratory cross/backcross protocol Aboagye-antwi et al.^15^. demonstrated evidence of involvement of the X island in reproductive isolation in the form of short-range assortive mating in laboratory-induced mixed-recombinant swarms^15^. Findings from these studies of laboratory-produced recombinants, naturally occurring recombinants and spatial swarm segregation, show a strong association between assortative mating mechanisms and the X island of speciation. They also lend unprecedented support to island-of-speciation models of sympatric speciation facilitated by pericentric recombination suppression.

## Methods

### Study sites

Vallée du Kou is a rice-growing area developed in the early 1970s in Western Burkina Faso about 30 km North of Bobo-Dioulasso (11° 22′ 26″ N, 4° 24′ 42″ W). It contains seven villages totalling 4470 habitants. The survey was conducted in VK7 (11° 24′ 17″ N, 4° 24′ 54″ W), located on the boundary of the rice field and cotton cultivation area characterized by wooded savannah. The mean annual rainfall is about 1200 mm and the area is characterized by a rainy season from May to October and a dry season from November to April. The river Kou is a permanent source of irrigation and there are 2 rice crops per year (January–May and July–November). Because of the irrigation system, the rice fields form permanent mosquito breeding sites that are preferentially colonized by *An. coluzzii*^26,28,41^. During the rainy season, additional rainy-dependent breeding sites are very often found in depressions and ponds, allowing the development of *An. gambiae*.

Soumousso (11° 00′ 46″ N, 4° 02′ 45″ W) and Bana (12° 36′ 00″ N, 3° 28′ 59″ W) are villages located in the humid savannah of Western Burkina Faso. As in VK7 the rainy season occurs from May to October followed by a long dry season from November to April. The average annual rainfall ranges from 1000 to 1200 mm. In Soumousso both incipient species coexist in sympatry and their highest density occurs in September. However, the relative frequencies of the two species change over time with *An. gambiae* being predominant from July to November and *An. coluzzii* from December to June. *An. funestus, An. arabiensis* and *An. nili* are also found at lower densities^41^. The village of Bana is characterized by comparable seasonality but more balanced densities of *An. coluzzii* and *An. gambiae* swarming males^26^ even though *An. coluzzii* dominated largely the indoor resting fauna of the complex An. gambiae over a two-year period of collection in this village^64^*.*

### Swarming mosquito collections

A survey of swarms was undertaken by trained observers in the three study sites: VK7, Soumousso and Bana. Observations were made to identify swarm sites scattered throughout the entire villages that were used every evening. Once located, these swarms were sampled every month from July to November in 2006, 2007, 2008 and 2011 in Soumousso, 2006, 2008 and 2011 in VK7 and 2012 in Bana. In order to catch a substantial number of females from swarms largely dominated by males, two captors spent the first 15 min after swarm initiation (first male arriving) capturing mating pairs as they formed, fell or flew out of the swarm. The whole swarm was then collected. Mating pairs and swarms were sampled using an insect net then aspirated into individual swarm cups as described previously^25,41,65^. Collected mosquitoes, including swarming males, females and mating pairs were respectively aspirated into separate cups and tubes, then killed with ethylic ether, identified morphologically as *An. gambiae* s.l.^66^ and kept in 70% ethanol in 1.5 ml tubes before being genotyped for subsequent DNA analysis.

### Assignation of swarming mosquitoes to the three types of swarms

Mosquito swarms were characterized based on the PCR results which includes only one SNP in the X-chromosome as described previously^26,67,68^. Swarms are considered as pure types for one species if they are exclusively comprised of individuals of that species. Otherwise, swarms are considered as mixed if they are composed of individuals of both species regardless of the constituent proportions. Then mosquitoes identified at molecular level were assigned to the three types of swarms including *An. gambiae* pure type, *An. coluzzii* pure type or both species mixed type (Table 1).

### Resting adult collections and larvae sampling

Except for the village of Bana and VK7 in 2007 females, indoor resting males, and females of *An. gambiae* s.l. were collected in each of villages during the same time periods of the swarm monitoring, using insecticide indoor spray catch. Resting females and males from different houses were aspirated into separate cups, killed with ethylic ether, identified morphologically as *An. gambiae* s.l.^66^ and kept in 70% ethanol in 1.5 ml tubes for subsequent DNA analysis.

In some years and for some locations, *Anopheles gambiae* s.l. larvae were also collected from larval habitats including footprint, puddle, pond, and rice field available during the same time periods as the swarm monitoring and indoor spray catch. All collected mosquito larvae were examined carefully, anopheline were separated from culicine larvae for classification into 1st to 4th instars, then immediate preservation was carried out in 70% ethanol for subsequent DNA analysis.

### Mosquito identification by polymerase chain reaction (PCR)

DNA was extracted from whole bodies of adult mosquitoes identified as *An. gambiae* s.l., except for females whose abdomens were excluded to avoid the risk of contamination with DNA from sperm in the spermathecae. Individual DNA extractions were made using a standard protocol^25^ and were diagnosed to species level using the PCR developed by Santolamazza et al*.*^68^ and confirmed by PCR–RFLP (Restriction Fragment Length Polymorphism) developed by Fanello et al.^67^.

DNA was extracted from individual larvae using a standard protocol^25^. As hybrid males have a single copy of the hemizygous X chromosome, they cannot be distinguished from females using classic molecular diagnostics based on rDNA polymorphisms on that chromosome. Consequently, all larvae were sexed and genotyped to sibling species level using a single multiplex PCR as described previously that combined the Y-chromosome specific primers used for sperm detection elsewhere^69^ with primers targeting the insertion of a SINE (Short Interspersed Element) located in division 6 of the X-chromosome used in the species identification PCR developed by Santolamazza et al.^68^. The primers (S200X6-1F 5′-TCGCCTTAGACCTTGCGTTA-3′; S200X6-1R 5′-CGCTTCAAGAATTCGAGATAC-3′; S23-F 5′-CAAAACGACAGCAGTTCC-3′; S23-R 5′-TAAACCAAGTCCGTCGCT-3′) were combined into a single reaction after checking for possible dimmer problems using the tool available at http://www.thermoscientificbio.com/webtools/multipleprimer/. A PCR mixture contained 2 µl template DNA, 1.5 mM MgCl_2_, 0.5 µl of 5× buffer, 0.2 mM of each dNTPs, 1.5 pmol of each S200X6.1 primers, 2 pmol of each S23 primers and 0.05 units of Taq polymerase for a total volume of 25 µl was used. PCR reactions were performed on a Bio-Rad S1000™ Thermocycler with an initial denaturation at 94 °C for 10 min, followed by 35 cycles of 94 °C for 30 s, 54 °C for 30 s, and 72 °C for 1 min, followed by a final elongation step at 72 °C for 10 min. The PCR products were separated by gel electrophoresis to generate diagnostic sex and species-specific banding phenotypes^26^.

### Mosquito divergent island SNP (DIS) genotyping

Mosquitoes identified as *An. coluzzii* and *An. gambiae* were sent to LGC Ltd for Competitive Allele Specific PCR (KASP) genotyping using the same DIS loci used in previous surveys of hybridization and introgression between the sibling species^55,56^. The panel features 7 SNPs located in the X-chromosome island, 5 in the 2L and 3 in the 3L that are fixed differences between *An. coluzzii* or *An. gambiae* in absence of introgression^56^. DIS genotypes were used to distinguish non-hybrid individuals homozygous at all alleles specific to either *An. coluzzii* or *An. gambiae*, from F_1_ hybrids (heterozygous at all X, 2L and 3L loci in females, and 2L and 3L loci in hemizygous males), and from F_1+n_ backcrosses (characterized by recombinant genotypes). Backcrosses were assigned to *An. coluzzii* or *An. gambiae* based on most of the alleles specific to each sibling species present in their genotypes using a simple majority rule^21,55,56^. Additionally, a subset of samples was also genotyped at the L1014F West African and L1014S East African *kdr* mutations both located within the 2L island^70,71^. Except for analysed focusing on the *kdr* genetic sweep, individuals whose DIS genotypes were incomplete (missing SNP data) were not used in further analyses.

### Statistical analyses

All statistical analyses were performed using the software JMP 10 (SAS Institute, Inc). Frequencies of recombinant genotypes were analysed through multivariate logistic regression (Likelihood ratios) and the statistical significance of pairwise comparisons established by Likelihood Ratio test on odds ratios.

## Supplementary Information

Supplementary Table S1.

Supplementary Table S2.

Supplementary Table S3.

Supplementary Table S4.

Supplementary Table S5.

Supplementary Table S6.

Supplementary Table S7.

Supplementary Table S8.

Supplementary Table S9.

## Data Availability

The datasets generated during the current study are available from the corresponding authors on reasonable request.
