# Supplementary material for: Perfect association between spatial swarm segregation and the X-chromosome speciation island in hybridizing Anopheles coluzzii and Anopheles gambiae populations
Source: Sci Rep. 2022 Jun 24;12:10800. doi: 10.1038/s41598-022-14865-9 (PMC9232630; doi:10.1038/s41598-022-14865-9)
Supplement: Supplementary file 1 — Supplementary Table S1. [file 41598_2022_14865_MOESM1_ESM.docx]

**Table S1. DIS genotypes of males collected in swarms -** The number of individuals with given genotypes (count) and total number of individuals genotyped (N Total) per swarm type, sampling location, and year of collection are indicated. The two males with X-chromosomes non-matching that the swarm type they were captured from are shown in yellow lettering, the two hybrid male genotypes detected in red. DIS loci are described through their base-pair position (red positions are closest to centromeres) and chromosomal division. For each locus, homozygous (or hemizygous for X in males) genotypes characteristic of *An. coluzzii* are shaded in light blue and those of *An. gambiae.* in dark blue, heterozygous genotypes in yellow

|  | | | | | Chromosome X | | | | | | | Chromosome 2L | | | | | Chromosome 3L | | |
| --- | --- | --- | --- | --- | --- | --- | --- | --- | --- | --- | --- | --- | --- | --- | --- | --- | --- | --- | --- |
| Sampling location | | Swarm characteristics | | | 5D | 6 | | | | | | 20A | 20B | 20C | | | 38A | | |
| Locality | Year | Type | Count | N Total | 20015634 | 22105429 | 22105860 | 22497157 | 22750432 | 22750572 | 22944682 | 209536 | 1274353 | 2430786 | 2430915 | 2431005 | 296897 | 387877 | 413944 |
| Bana | 2012 | *An. coluzzii* | 1 | 65 | C | A | T | A | G | G | T | C:C | A:A | C:C | A:A | C:C | G:G | G:G | T:T |
| Bana | 2012 | *An. coluzzii* | 1 | 65 | C | A | T | A | G | G | T | C:C | A:A | T:C | G:A | T:C | G:G | G:G | T:T |
| Bana | 2012 | *An. coluzzii* | 1 | 65 | C | A | T | A | G | G | T | C:C | G:G | T:T | G:G | T:T | G:G | G:G | T:T |
| Bana | 2012 | *An. coluzzii* | 3 | 65 | C | A | T | A | G | G | T | C:C | A:G | T:C | G:A | T:C | G:G | G:G | T:T |
| Bana | 2012 | *An. coluzzii* | 1 | 65 | C | A | T | A | G | G | T | C:C | A:G | T:T | G:G | T:T | G:G | G:G | T:T |
| Bana | 2012 | *An. coluzzii* | 4 | 65 | C | A | T | A | G | G | T | T:C | G:G | T:T | G:G | T:T | G:G | G:G | T:T |
| Bana | 2012 | *An. coluzzii* | 12 | 65 | C | A | T | A | G | G | T | T:C | A:G | T:C | G:A | T:C | G:G | G:G | T:T |
| Bana | 2012 | *An. coluzzii* | 1 | 65 | C | A | T | A | G | G | T | T:C | A:G | T:T | G:G | T:T | G:G | G:G | T:T |
| Bana | 2012 | *An. coluzzii* | 41 | 65 | C | A | T | A | G | G | T | T:T | G:G | T:T | G:G | T:T | G:G | G:G | T:T |
| Bana | 2012 | *An. gambiae* | 57 | 57 | A | T | C | G | A | T | G | T:T | G:G | T:T | G:G | T:T | A:A | A:A | C:C |
| Soumousso | 2006 | *An. gambiae* | 2 | 28 | A | T | C | G | A | T | G | T:T | G:G | T:T | G:G | T:T | G:A | G:A | T:C |
| Soumousso | 2006 | *An. gambiae* | 26 | 28 | A | T | C | G | A | T | G | T:T | G:G | T:T | G:G | T:T | A:A | A:A | C:C |
| Soumousso | 2007 | *An. coluzzii* | 30 | 36 | C | A | T | A | G | G | T | C:C | A:A | C:C | A:A | C:C | G:G | G:G | T:T |
| Soumousso | 2007 | *An. coluzzii* | 1 | 36 | C | A | T | A | G | G | T | C:C | A:A | T:C | G:A | T:C | G:G | G:G | T:T |
| Soumousso | 2007 | *An. coluzzii* | 5 | 36 | C | A | T | A | G | G | T | T:C | A:G | T:C | G:A | T:C | G:G | G:G | T:T |
| Soumousso | 2007 | *An. gambiae* | 2 | 374 | C | A | T | A | G | G | T | T:T | G:G | T:T | G:G | T:T | A:A | A:A | C:C |
| Soumousso | 2007 | *An. gambiae* | 1 | 374 | A | T | C | G | A | T | G | T:C | A:G | T:T | G:G | T:T | A:A | A:A | C:C |
| Soumousso | 2007 | *An. gambiae* | 1 | 374 | A | T | C | G | A | T | G | T:T | G:G | T:T | G:G | T:T | G:G | G:G | T:T |
| Soumousso | 2007 | *An. gambiae* | 14 | 374 | A | T | C | G | A | T | G | T:T | G:G | T:T | G:G | T:T | G:A | G:A | T:C |
| Soumousso | 2007 | *An. gambiae* | 356 | 374 | A | T | C | G | A | T | G | T:T | G:G | T:T | G:G | T:T | A:A | A:A | C:C |
| Soumousso | 2008 | *An. gambiae* | 1 | 176 | A | T | C | G | A | T | G | T:C | A:G | T:C | G:A | T:C | G:A | G:A | T:C |
| Soumousso | 2008 | *An. gambiae* | 10 | 176 | A | T | C | G | A | T | G | T:T | G:G | T:T | G:G | T:T | G:A | G:A | T:C |
| Soumousso | 2008 | *An. gambiae* | 1 | 176 | A | T | C | G | A | T | G | T:T | G:G | T:T | G:G | T:T | G:A | A:A | C:C |
| Soumousso | 2008 | *An. gambiae* | 164 | 176 | A | T | C | G | A | T | G | T:T | G:G | T:T | G:G | T:T | A:A | A:A | C:C |
| Soumousso | 2011 | *An. coluzzii* | 1 | 15 | C | A | T | A | G | G | T | C:C | A:A | C:C | A:A | C:C | G:G | G:G | T:T |
| Soumousso | 2011 | *An. coluzzii* | 1 | 15 | C | A | T | A | G | G | T | C:C | A:G | T:C | G:A | T:C | G:G | G:G | T:T |
| Soumousso | 2011 | *An. coluzzii* | 1 | 15 | C | A | T | A | G | G | T | T:C | G:G | T:C | G:A | T:C | G:G | G:G | T:T |
| Soumousso | 2011 | *An. coluzzii* | 8 | 15 | C | A | T | A | G | G | T | T:C | A:G | T:C | G:A | T:C | G:G | G:G | T:T |
| Soumousso | 2011 | *An. coluzzii* | 1 | 15 | C | A | T | A | G | G | T | T:T | G:G | T:C | G:A | T:C | G:G | G:G | T:T |
| Soumousso | 2011 | *An. coluzzii* | 3 | 15 | C | A | T | A | G | G | T | T:T | G:G | T:T | G:G | T:T | G:G | G:G | T:T |
| Soumousso | 2011 | *An. gambiae* | 1 | 74 | A | T | C | G | A | T | G | T:T | G:G | T:T | G:G | T:T | G:A | G:A | T:C |
| Soumousso | 2011 | *An. gambiae* | 73 | 74 | A | T | C | G | A | T | G | T:T | G:G | T:T | G:G | T:T | A:A | A:A | C:C |
| VK7 | 2006 | *An. coluzzii* | 17 | 67 | C | A | T | A | G | G | T | C:C | A:A | C:C | A:A | C:C | G:G | G:G | T:T |
| VK7 | 2006 | *An. coluzzii* | 1 | 67 | C | A | T | A | G | G | T | T:C | A:A | C:C | A:A | C:C | G:G | G:G | T:T |
| VK7 | 2006 | *An. coluzzii* | 1 | 67 | C | A | T | A | G | G | T | T:C | G:G | T:C | G:A | T:C | G:G | G:G | T:T |
| VK7 | 2006 | *An. coluzzii* | 1 | 67 | C | A | T | A | G | G | T | T:C | A:G | T:C | A:A | T:C | G:G | G:G | T:T |
| VK7 | 2006 | *An. coluzzii* | 27 | 67 | C | A | T | A | G | G | T | T:C | A:G | T:C | G:A | T:C | G:G | G:G | T:T |
| VK7 | 2006 | *An. coluzzii* | 1 | 67 | C | A | T | A | G | G | T | T:C | A:G | T:T | G:G | T:T | G:G | G:G | T:T |
| VK7 | 2006 | *An. coluzzii* | 17 | 67 | C | A | T | A | G | G | T | T:T | G:G | T:T | G:G | T:T | G:G | G:G | T:T |
| VK7 | 2006 | *An. coluzzii* | 2 | 67 | C | A | T | A | G | G | T | T:T | A:G | T:C | G:A | T:C | G:G | G:G | T:T |
| VK7 | 2006 | *An. gambiae* | 1 | 16 | A | T | C | G | A | T | G | T:T | G:G | T:T | G:G | T:T | G:A | G:A | T:C |
| VK7 | 2006 | *An. gambiae* | 15 | 16 | A | T | C | G | A | T | G | T:T | G:G | T:T | G:G | T:T | A:A | A:A | C:C |
| VK7 | 2008 | *An. coluzzii* | 9 | 493 | C | A | T | A | G | G | T | C:C | A:A | C:C | A:A | C:C | G:G | G:G | T:T |
| VK7 | 2008 | *An. coluzzii* | 2 | 493 | C | A | T | A | G | G | T | C:C | A:A | T:C | G:A | T:C | G:G | G:G | T:T |
| VK7 | 2008 | *An. coluzzii* | 3 | 493 | C | A | T | A | G | G | T | C:C | A:G | T:C | G:A | T:C | G:G | G:G | T:T |
| VK7 | 2008 | *An. coluzzii* | 3 | 493 | C | A | T | A | G | G | T | T:C | G:G | T:C | G:A | T:C | G:G | G:G | T:T |
| VK7 | 2008 | *An. coluzzii* | 30 | 493 | C | A | T | A | G | G | T | T:C | G:G | T:T | G:G | T:T | G:G | G:G | T:T |
| VK7 | 2008 | *An. coluzzii* | 2 | 493 | C | A | T | A | G | G | T | T:C | A:G | C:C | A:A | C:C | G:G | G:G | T:T |
| VK7 | 2008 | *An. coluzzii* | 1 | 493 | C | A | T | A | G | G | T | T:C | A:G | T:C | A:A | T:C | G:G | G:G | T:T |
| VK7 | 2008 | *An. coluzzii* | 91 | 493 | C | A | T | A | G | G | T | T:C | A:G | T:C | G:A | T:C | G:G | G:G | T:T |
| VK7 | 2008 | *An. coluzzii* | 13 | 493 | C | A | T | A | G | G | T | T:C | A:G | T:T | G:G | T:T | G:G | G:G | T:T |
| VK7 | 2008 | *An. coluzzii* | 5 | 493 | C | A | T | A | G | G | T | T:T | G:G | T:C | G:A | T:C | G:G | G:G | T:T |
| VK7 | 2008 | *An. coluzzii* | 328 | 493 | C | A | T | A | G | G | T | T:T | G:G | T:T | G:G | T:T | G:G | G:G | T:T |
| VK7 | 2008 | *An. coluzzii* | 1 | 493 | C | A | T | A | G | G | T | T:T | G:G | T:T | G:G | T:T | G:A | G:A | T:C |
| VK7 | 2008 | *An. coluzzii* | 5 | 493 | C | A | T | A | G | G | T | T:T | A:G | T:C | G:A | T:C | G:G | G:G | T:T |
| VK7 | 2011 | *An. coluzzii* | 9 | 622 | C | A | T | A | G | G | T | C:C | A:A | C:C | A:A | C:C | G:G | G:G | T:T |
| VK7 | 2011 | *An. coluzzii* | 2 | 622 | C | A | T | A | G | G | T | C:C | A:A | T:C | G:A | T:C | G:G | G:G | T:T |
| VK7 | 2011 | *An. coluzzii* | 1 | 622 | C | A | T | A | G | G | T | C:C | G:G | T:T | G:G | T:T | G:G | G:G | T:T |
| VK7 | 2011 | *An. coluzzii* | 6 | 622 | C | A | T | A | G | G | T | C:C | A:G | T:C | G:A | T:C | G:G | G:G | T:T |
| VK7 | 2011 | *An. coluzzii* | 2 | 622 | C | A | T | A | G | G | T | T:C | G:G | T:C | G:A | T:C | G:G | G:G | T:T |
| VK7 | 2011 | *An. coluzzii* | 46 | 622 | C | A | T | A | G | G | T | T:C | G:G | T:T | G:G | T:T | G:G | G:G | T:T |
| VK7 | 2011 | *An. coluzzii* | 87 | 622 | C | A | T | A | G | G | T | T:C | A:G | T:C | G:A | T:C | G:G | G:G | T:T |
| VK7 | 2011 | *An. coluzzii* | 1 | 622 | C | A | T | A | G | G | T | T:C | A:G | T:C | G:A | T:C | G:A | G:A | T:C |
| VK7 | 2011 | *An. coluzzii* | 26 | 622 | C | A | T | A | G | G | T | T:C | A:G | T:T | G:G | T:T | G:G | G:G | T:T |
| VK7 | 2011 | *An. coluzzii* | 22 | 622 | C | A | T | A | G | G | T | T:T | G:G | T:C | G:A | T:C | G:G | G:G | T:T |
| VK7 | 2011 | *An. coluzzii* | 418 | 622 | C | A | T | A | G | G | T | T:T | G:G | T:T | G:G | T:T | G:G | G:G | T:T |
| VK7 | 2011 | *An. coluzzii* | 2 | 622 | C | A | T | A | G | G | T | T:T | A:G | T:C | G:A | T:C | G:G | G:G | T:T |
