# Supplementary material for: Perfect association between spatial swarm segregation and the X-chromosome speciation island in hybridizing Anopheles coluzzii and Anopheles gambiae populations
Source: Sci Rep. 2022 Jun 24;12:10800. doi: 10.1038/s41598-022-14865-9 (PMC9232630; doi:10.1038/s41598-022-14865-9)
Supplement: Supplementary file 5 — Supplementary Table S5. [file 41598_2022_14865_MOESM5_ESM.docx]

**Table S5. DIS of female larvae collected from breeding sites -** The number of individuals with given genotypes (count) and total number of individuals of each species genotyped per larval breeding site, sampling location, and year of collection are indicated. DIS loci are described through their base-pair position (red positions are loci closest to the centromeres) and chromosomal division. For each locus, homozygous genotypes characteristic of *An. coluzzii* are shaded in light blue and those of *An. gambiae* in dark blue, heterozygous genotypes in yellow.

|  |  |  | |  | | Chromosome X | | | | | | | | Chromosome 2L | | | | | Chromosome 3L | | | |
| --- | --- | --- | --- | --- | --- | --- | --- | --- | --- | --- | --- | --- | --- | --- | --- | --- | --- | --- | --- | --- | --- | --- |
| Sampling location | | Sample | | | | | 5D | 6 | | | | | | 20A | 20B | 20C | | | | 38A | | |
| Locality | Year | Species | Count | | N Total | | 20015634 | 22105429 | 22105860 | 22497157 | 22750432 | 22750572 | 22944682 | 209536 | 1274353 | 2430786 | 2430915 | 2431005 | | 296897 | 387877 | 413944 |
| Soumousso | 2006 | *An. coluzzii* | 2 | | 49 | | C:C | A:A | T:T | A:A | G:G | G:G | T:T | C:C | A:A | C:C | A:A | C:C | | G:G | G:G | T:T |
| Soumousso | 2006 | *An. coluzzii* | 1 | | 49 | | C:C | A:A | T:T | A:A | G:G | G:G | T:T | C:C | A:A | T:C | G:A | T:C | | G:G | G:G | T:T |
| Soumousso | 2006 | *An. coluzzii* | 1 | | 49 | | C:C | A:A | T:T | A:A | G:G | G:G | T:T | T:C | A:G | T:C | G:A | T:C | | G:G | G:G | T:T |
| Soumousso | 2006 | *An. gambiae* | 45 | | 49 | | A:A | T:T | C:C | G:G | A:A | T:T | G:G | T:T | G:G | T:T | G:G | T:T | | A:A | A:A | C:C |
| Soumousso | 2008 | *An. coluzzii* | 1 | | 56 | | C:C | A:A | T:T | A:A | G:G | G:G | T:T | T:C | A:G | T:C | G:A | T:C | | G:G | G:G | T:T |
| Soumousso | 2008 | *An. gambiae* | 5 | | 56 | | A:A | T:T | C:C | G:G | A:A | T:T | G:G | T:T | G:G | T:T | G:G | T:T | | G:A | G:A | T:C |
| Soumousso | 2008 | *An. gambiae* | 50 | | 56 | | A:A | T:T | C:C | G:G | A:A | T:T | G:G | T:T | G:G | T:T | G:G | T:T | | A:A | A:A | C:C |
| Soumousso | 2011 | *An. coluzzii* | 1 | | 64 | | C:C | A:A | T:T | A:A | G:G | G:G | T:T | C:C | A:A | C:C | A:A | C:C | | G:G | G:G | T:T |
| Soumousso | 2011 | *An. coluzzii* | 3 | | 64 | | C:C | A:A | T:T | A:A | G:G | G:G | T:T | T:C | A:G | T:C | G:A | T:C | | G:G | G:G | T:T |
| Soumousso | 2011 | *An. coluzzii* | 1 | | 64 | | C:C | A:A | T:T | A:A | G:G | G:G | T:T | T:C | A:G | T:T | G:G | T:T | | G:G | G:G | T:T |
| Soumousso | 2011 | *An. coluzzii* | 4 | | 64 | | C:C | A:A | T:T | A:A | G:G | G:G | T:T | T:T | G:G | T:T | G:G | T:T | | G:G | G:G | T:T |
| Soumousso | 2011 | *An. gambiae* | 2 | | 64 | | A:A | T:T | C:C | G:G | A:A | T:T | G:G | T:T | G:G | T:T | G:G | T:T | | G:A | G:A | T:C |
| Soumousso | 2011 | *An. gambiae* | 53 | | 64 | | A:A | T:T | C:C | G:G | A:A | T:T | G:G | T:T | G:G | T:T | G:G | T:T | | A:A | A:A | C:C |
| VK7 | 2011 | *An. coluzzii* | 1 | | 41 | | C:C | A:A | T:T | A:A | G:G | G:G | T:T | C:C | A:A | C:C | A:A | C:C | | G:G | G:G | T:T |
| VK7 | 2011 | *An. coluzzii* | 1 | | 41 | | C:C | A:A | T:T | A:A | G:G | G:G | T:T | C:C | G:G | T:T | G:G | T:T | | G:G | G:G | T:T |
| VK7 | 2011 | *An. coluzzii* | 2 | | 41 | | C:C | A:A | T:T | A:A | G:G | G:G | T:T | C:C | A:G | T:C | G:A | T:C | | G:G | G:G | T:T |
| VK7 | 2011 | *An. coluzzii* | 2 | | 41 | | C:C | A:A | T:T | A:A | G:G | G:G | T:T | T:C | G:G | T:T | G:G | T:T | | G:G | G:G | T:T |
| VK7 | 2011 | *An. coluzzii* | 1 | | 41 | | C:C | A:A | T:T | A:A | G:G | G:G | T:T | T:C | A:G | C:C | A:A | C:C | | G:G | G:G | T:T |
| VK7 | 2011 | *An. coluzzii* | 4 | | 41 | | C:C | A:A | T:T | A:A | G:G | G:G | T:T | T:C | A:G | T:C | G:A | T:C | | G:G | G:G | T:T |
| VK7 | 2011 | *An. coluzzii* | 3 | | 41 | | C:C | A:A | T:T | A:A | G:G | G:G | T:T | T:C | A:G | T:T | G:G | T:T | | G:G | G:G | T:T |
| VK7 | 2011 | *An. coluzzii* | 27 | | 41 | | C:C | A:A | T:T | A:A | G:G | G:G | T:T | T:T | G:G | T:T | G:G | T:T | | G:G | G:G | T:T |
