# Supplementary material for: Perfect association between spatial swarm segregation and the X-chromosome speciation island in hybridizing Anopheles coluzzii and Anopheles gambiae populations
Source: Sci Rep. 2022 Jun 24;12:10800. doi: 10.1038/s41598-022-14865-9 (PMC9232630; doi:10.1038/s41598-022-14865-9)
Supplement: Supplementary file 6 — Supplementary Table S6. [file 41598_2022_14865_MOESM6_ESM.docx]

**Table S6. DIS from males collected from the Indoor Residual Fauna (IRF) -** The number of individuals with given genotypes (Count) and total number of individuals (N Total) of each species genotyped per sampling location, and year of collection are indicated. DIS loci are described through their base-pair position (red positions are closest to centromeres) and chromosomal division. For each locus, homozygous (or hemizygous for X in males) genotypes characteristic of *An. coluzzii* are shaded in light blue and those of *An. gambiae* in dark blue, heterozygous genotypes in yellow.

|  |  |  |  | | | Chromosome X | | | | | | | Chromosome 2L | | | | | | | Chromosome 3L | | |
| --- | --- | --- | --- | --- | --- | --- | --- | --- | --- | --- | --- | --- | --- | --- | --- | --- | --- | --- | --- | --- | --- | --- |
| Sampling location | | Sample | | | | 5D | 6 | | | | | | 20A | 20B | | 20C | | | | 38A | | |
| Locality | Year | species | | Count | N Total | 20015634 | 22105429 | 22105860 | 22497157 | 22750432 | 22750572 | 22944682 | 209536 | | 1274353 | 2430786 | 2430915 | 2431005 | 296897 | | 387877 | 413944 |
| Soumousso | 2007 | *An. coluzzii* | | 3 | 65 | C | A | T | A | G | G | T | C:C | | A:A | C:C | A:A | C:C | G:G | | G:G | T:T |
| Soumousso | 2007 | *An. gambiae* | | 62 | 65 | A | T | C | G | A | T | G | T:T | | G:G | T:T | G:G | T:T | A:A | | A:A | C:C |
| Soumousso | 2008 | *An. coluzzii* | | 2 | 37 | C | A | T | A | G | G | T | C:C | | A:A | C:C | A:A | C:C | G:G | | G:G | T:T |
| Soumousso | 2008 | *An. coluzzii* | | 1 | 37 | C | A | T | A | G | G | T | T:C | | G:G | T:T | G:G | T:T | G:G | | G:G | T:T |
| Soumousso | 2008 | *An. coluzzii* | | 1 | 37 | C | A | T | A | G | G | T | T:C | | A:G | T:C | G:A | T:C | G:G | | G:G | T:T |
| Soumousso | 2008 | *An. gambiae* | | 2 | 37 | A | T | C | G | A | T | G | T:T | | G:G | T:T | G:G | T:T | G:A | | G:A | T:C |
| Soumousso | 2008 | *An. gambiae* | | 31 | 37 | A | T | C | G | A | T | G | T:T | | G:G | T:T | G:G | T:T | A:A | | A:A | C:C |
| Soumousso | 2011 | *An. coluzzii* | | 2 | 40 | C | A | T | A | G | G | T | T:C | | A:G | T:C | G:A | T:C | G:G | | G:G | T:T |
| Soumousso | 2011 | *An. coluzzii* | | 1 | 40 | C | A | T | A | G | G | T | T:T | | G:G | T:C | G:A | T:C | G:G | | G:G | T:T |
| Soumousso | 2011 | *An. coluzzii* | | 1 | 40 | C | A | T | A | G | G | T | T:T | | G:G | T:T | G:G | T:T | G:G | | G:G | T:T |
| Soumousso | 2011 | *An. gambiae* | | 2 | 40 | A | T | C | G | A | T | G | T:T | | G:G | T:T | G:G | T:T | G:A | | G:A | T:C |
| Soumousso | 2011 | *An. gambiae* | | 34 | 40 | A | T | C | G | A | T | G | T:T | | G:G | T:T | G:G | T:T | A:A | | A:A | C:C |
| Soumousso | 2012 | *An. gambiae* | | 30 | 30 | A | T | C | G | A | T | G | T:T | | G:G | T:T | G:G | T:T | A:A | | A:A | C:C |
| VK7 | 2006 | *An. coluzzii* | | 20 | 93 | C | A | T | A | G | G | T | C:C | | A:A | C:C | A:A | C:C | G:G | | G:G | T:T |
| VK7 | 2006 | *An. coluzzii* | | 2 | 93 | C | A | T | A | G | G | T | T:C | | G:G | T:T | G:G | T:T | G:G | | G:G | T:T |
| VK7 | 2006 | *An. coluzzii* | | 19 | 93 | C | A | T | A | G | G | T | T:C | | A:G | T:C | G:A | T:C | G:G | | G:G | T:T |
| VK7 | 2006 | *An. coluzzii* | | 13 | 93 | C | A | T | A | G | G | T | T:T | | G:G | T:T | G:G | T:T | G:G | | G:G | T:T |
| VK7 | 2006 | *An. gambiae* | | 39 | 93 | A | T | C | G | A | T | G | T:T | | G:G | T:T | G:G | T:T | A:A | | A:A | C:C |
| VK7 | 2008 | *An. coluzzii* | | 1 | 17 | C | A | T | A | G | G | T | C:C | | A:A | C:C | A:A | C:C | G:G | | G:G | T:T |
| VK7 | 2008 | *An. coluzzii* | | 3 | 17 | C | A | T | A | G | G | T | T:C | | A:G | T:C | G:A | T:C | G:G | | G:G | T:T |
| VK7 | 2008 | *An. coluzzii* | | 12 | 17 | C | A | T | A | G | G | T | T:T | | G:G | T:T | G:G | T:T | G:G | | G:G | T:T |
| VK7 | 2008 | *An. gambiae* | | 1 | 17 | A | T | C | G | A | T | G | T:T | | G:G | T:T | G:G | T:T | A:A | | A:A | C:C |
| VK7 | 2011 | *An. coluzzii* | | 1 | 48 | C | A | T | A | G | G | T | C:C | | A:A | C:C | A:A | C:C | G:G | | G:G | T:T |
| VK7 | 2011 | *An. coluzzii* | | 1 | 48 | C | A | T | A | G | G | T | C:C | | G:G | T:T | G:G | T:T | G:G | | G:G | T:T |
| VK7 | 2011 | *An. coluzzii* | | 5 | 48 | C | A | T | A | G | G | T | T:C | | G:G | T:T | G:G | T:T | G:G | | G:G | T:T |
| VK7 | 2011 | *An. coluzzii* | | 7 | 48 | C | A | T | A | G | G | T | T:C | | A:G | T:C | G:A | T:C | G:G | | G:G | T:T |
| VK7 | 2011 | *An. coluzzii* | | 2 | 48 | C | A | T | A | G | G | T | T:C | | A:G | T:T | G:G | T:T | G:G | | G:G | T:T |
| VK7 | 2011 | *An. coluzzii* | | 3 | 48 | C | A | T | A | G | G | T | T:T | | G:G | T:C | G:A | T:C | G:G | | G:G | T:T |
| VK7 | 2011 | *An. coluzzii* | | 19 | 48 | C | A | T | A | G | G | T | T:T | | G:G | T:T | G:G | T:T | G:G | | G:G | T:T |
| VK7 | 2011 | *An. gambiae* | | 10 | 48 | A | T | C | G | A | T | G | T:T | | G:G | T:T | G:G | T:T | A:A | | A:A | C:C |
| VK7 | 2012 | *An. coluzzii* | | 1 | 24 | C | A | T | A | G | G | T | C:C | | A:A | T:C | G:A | T:C | G:G | | G:G | T:T |
| VK7 | 2012 | *An. coluzzii* | | 1 | 24 | C | A | T | A | G | G | T | C:C | | A:G | T:C | G:A | T:C | G:G | | G:G | T:T |
| VK7 | 2012 | *An. coluzzii* | | 1 | 24 | C | A | T | A | G | G | T | T:C | | A:G | C:C | A:A | C:C | G:G | | G:G | T:T |
| VK7 | 2012 | *An. coluzzii* | | 2 | 24 | C | A | T | A | G | G | T | T:C | | A:G | T:C | G:A | T:C | G:G | | G:G | T:T |
| VK7 | 2012 | *An. coluzzii* | | 19 | 24 | C | A | T | A | G | G | T | T:T | | G:G | T:T | G:G | T:T | G:G | | G:G | T:T |
