# Supplementary material for: Perfect association between spatial swarm segregation and the X-chromosome speciation island in hybridizing Anopheles coluzzii and Anopheles gambiae populations
Source: Sci Rep. 2022 Jun 24;12:10800. doi: 10.1038/s41598-022-14865-9 (PMC9232630; doi:10.1038/s41598-022-14865-9)
Supplement: Supplementary file 8 — Supplementary Table S8. [file 41598_2022_14865_MOESM8_ESM.docx]

**Table S8.** **Number and percentages of recombined chromosome islands -** the number of pericentric islands with evidence of recombination between SNP markers is shown for each chromosome in males and females collected from swarms and from swarms, larval and adult indoor resting collections combined.

| **Life stage** | | **Species** | **Sex** | **Recombination within island** | | |
| --- | --- | --- | --- | --- | --- | --- |
|  |  | |  | **X** | **2L** | **3L** |
|  |  | |  | *N*/Total (%) | *N*/Total (%) | *N*/Total (%) |
| Swarms | *An. coluzzii* | | Males | 0/1332 | 197/2664 (7.39) | 0/2664 |
|  |  | | Females | 0/386 | 23/386 (5.96) | 0/386 |
|  | *An. gambiae* | | Males | 0/729 | 1/1458 (0.07) | 1/1458 (0.07) |
|  |  | | Females | 0/146 | 0/146 | 0/146 |
| Total swarms | - | | - | 0/2593 | 221/4654 (4.75) | 1/4654 (0.02) |
| All | *An. coluzzii* | | Males | 0/1533 | 227/3066 (7.40) | 0/3066 |
|  |  | | Females | 0/856 | 60/856 (7.0) | 0/856 |
|  | *An. gambiae* | | Males | 0/1063 | 1/2126 (0.05) | 1/2126 (0.05) |
|  |  | | Females | 0/808 | 0/808 | 1/808 (0.12) |
| Total All | - | | - | 0/4260 | 288/6856 (4.20) | 2/6856 (0.03) |
