# Supplementary material for: Perfect association between spatial swarm segregation and the X-chromosome speciation island in hybridizing Anopheles coluzzii and Anopheles gambiae populations
Source: Sci Rep. 2022 Jun 24;12:10800. doi: 10.1038/s41598-022-14865-9 (PMC9232630; doi:10.1038/s41598-022-14865-9)
Supplement: Supplementary file 9 — Supplementary Table S9. [file 41598_2022_14865_MOESM9_ESM.docx]

**Table S9.** **DIS genotypes of all individuals characterized at knockdown *kdr* pesticide resistance locus located within the 2L island -** The number of individuals with given genotypes (count) and total number of individuals of each species (N Total) per sampling location, and year of collection are indicated. DIS loci are described through their base-pair position (red positions are loci closest to the centromeres) and chromosomal division. For each locus, genotypes characteristic of *An. coluzzii* are shaded in light blue and those of *An. gambiae* in dark blue, heterozygous genotypes in yellow. *Kdr* genotypes are highlighted in shades of red (dark red, red and pink are homozygous resistant, heterozygous resistant and susceptible genotypes).

|  |  |  |  |  | Chromosome X | | | | | | | Chromosome 2L | | | | | | Chromosome 3L | | |
| --- | --- | --- | --- | --- | --- | --- | --- | --- | --- | --- | --- | --- | --- | --- | --- | --- | --- | --- | --- | --- |
| Sampling Location | | Samples | | | 5D | 6 | | | | | | 20A | 20B | 20C | | | | 38A | | |
| Locality | Year | Species | Count | N Total | 20 015634 | 22105429 | 22105860 | 22497157 | 22750432 | 22750572 | 22944682 | 209536 | 1274353 | 2422652 | 2430786 | 2430915 | 2431005 | 296897 | 387877 | 413944 |
| Soumousso | 2006 | *An. coluzzii* | 8 | 94 | C:C | A:A | T:T | A:A | G:G | G:G | T:T | C:C | A:A | A:A | C:C | A:A | C:C | G:G | G:G | T:T |
|  | 2006 | *An. coluzzii* | 1 | 94 | C:C | A:A | T:T | A:A | G:G | G:G | T:T | C:C | A:A | A:A | T:C | G:A | T:C | G:G | G:G | T:T |
|  | 2006 | *An. coluzzii* | 1 | 94 | C:C | A:A | T:T | A:A | G:G | G:G | T:T | T:C | G:A | T:A | T:C | G:A | T:C | G:G | G:G | T:T |
|  | 2006 | *An. gambiae* | 1 | 94 | A:A | T:T | C:C | G:G | A:A | T:T | G:G | T:T | G:G | T:T | T:T | G:G | T:T | G:A | G:A | T:C |
|  | 2006 | *An. gambiae* | 7 | 94 | A:A | T:T | C:C | G:G | A:A | T:T | G:G | T:T | G:G | T:A | T:T | G:G | T:T | A:A | A:A | C:C |
|  | 2006 | *An. gambiae* | 76 | 94 | A:A | T:T | C:C | G:G | A:A | T:T | G:G | T:T | G:G | T:T | T:T | G:G | T:T | A:A | A:A | C:C |
|  | 2007 | *An. coluzzii* | 6 | 199 | C:C | A:A | T:T | A:A | G:G | G:G | T:T | C:C | A:A | A:A | C:C | A:A | C:C | G:G | G:G | T:T |
|  | 2007 | *An. coluzzii* | 2 | 199 | C:C | A:A | T:T | A:A | G:G | G:G | T:T | T:C | G:A | T:A | T:C | G:A | T:C | G:G | G:G | T:T |
|  | 2007 | *An. coluzzii* | 1 | 199 | C:C | A:A | T:T | A:A | G:G | G:G | T:T | T:T | G:G | T:A | T:T | G:G | T:T | A:A | A:A | C:C |
|  | 2007 | *An. coluzzii* | 1 | 199 | C:C | A:A | T:T | A:A | G:G | G:G | T:T | T:T | G:G | T:T | T:T | G:G | T:T | G:G | G:G | T:T |
|  | 2007 | *An. coluzzii* | 1 | 199 | C:C | A:A | T:T | A:A | G:G | G:G | T:T | T:T | G:G | T:T | T:T | G:G | T:T | A:A | A:A | C:C |
|  | 2007 | *An. gambiae* | 1 | 199 | A:A | T:T | C:C | G:G | A:A | T:T | G:G | T:T | G:G | A:A | T:T | G:G | T:T | G:A | G:A | T:C |
|  | 2007 | *An. gambiae* | 1 | 199 | A:A | T:T | C:C | G:G | A:A | T:T | G:G | T:T | G:G | T:A | T:T | G:G | T:T | G:A | G:A | T:C |
|  | 2007 | *An. gambiae* | 2 | 199 | A:A | T:T | C:C | G:G | A:A | T:T | G:G | T:T | G:G | T:T | T:T | G:G | T:T | G:A | G:A | T:C |
|  | 2007 | *An. gambiae* | 1 | 199 | A:A | T:T | C:C | G:G | A:A | T:T | G:G | T:T | G:G | T:T | T:T | G:G | T:T | G:A | A:A | C:C |
|  | 2007 | *An. gambiae* | 6 | 199 | A:A | T:T | C:C | G:G | A:A | T:T | G:G | T:T | G:G | A:A | T:T | G:G | T:T | A:A | A:A | C:C |
|  | 2007 | *An. gambiae* | 57 | 199 | A:A | T:T | C:C | G:G | A:A | T:T | G:G | T:T | G:G | T:A | T:T | G:G | T:T | A:A | A:A | C:C |
|  | 2007 | *An. gambiae* | 120 | 199 | A:A | T:T | C:C | G:G | A:A | T:T | G:G | T:T | G:G | T:T | T:T | G:G | T:T | A:A | A:A | C:C |
|  | 2008 | *An. coluzzii* | 7 | 398 | C:C | A:A | T:T | A:A | G:G | G:G | T:T | C:C | A:A | A:A | C:C | A:A | C:C | G:G | G:G | T:T |
|  | 2008 | *An. coluzzii* | 4 | 398 | C:C | A:A | T:T | A:A | G:G | G:G | T:T | T:C | G:A | T:A | T:C | G:A | T:C | G:G | G:G | T:T |
|  | 2008 | *An. coluzzii* | 1 | 398 | C:C | A:A | T:T | A:A | G:G | G:G | T:T | T:C | G:G | T:T | T:T | G:G | T:T | G:G | G:G | T:T |
|  | 2008 | *An. coluzzii* | 1 | 398 | C:C | A:A | T:T | A:A | G:G | G:G | T:T | T:T | G:G | T:T | T:T | G:G | T:T | G:G | G:G | T:T |
|  | 2008 | *An. gambiae* | 7 | 398 | A:A | T:T | C:C | G:G | A:A | T:T | G:G | T:T | G:G | T:A | T:T | G:G | T:T | G:A | G:A | T:C |
|  | 2008 | *An. gambiae* | 16 | 398 | A:A | T:T | C:C | G:G | A:A | T:T | G:G | T:T | G:G | T:T | T:T | G:G | T:T | G:A | G:A | T:C |
|  | 2008 | *An. gambiae* | 1 | 398 | A:A | T:T | C:C | G:G | A:A | T:T | G:G | T:T | G:G | T:T | T:T | G:G | T:T | G:A | A:A | C:C |
|  | 2008 | *An. gambiae* | 1 | 398 | A:A | T:T | C:C | G:G | A:A | T:T | G:G | T:T | G:G | A:A | T:T | G:G | T:T | A:A | A:A | C:C |
|  | 2008 | *An. gambiae* | 48 | 398 | A:A | T:T | C:C | G:G | A:A | T:T | G:G | T:T | G:G | T:A | T:T | G:G | T:T | A:A | A:A | C:C |
|  | 2008 | *An. gambiae* | 311 | 398 | A:A | T:T | C:C | G:G | A:A | T:T | G:G | T:T | G:G | T:T | T:T | G:G | T:T | A:A | A:A | C:C |
|  | 2008 | *An. gambiae* | 1 | 398 | A:A | T:T | C:C | G:G | A:A | T:T | G:G | T:C | G:A | T:A | T:C | G:A | T:C | G:A | G:A | T:C |
|  | 2011 | *An. coluzzii* | 2 | 277 | C:C | A:A | T:T | A:A | G:G | G:G | T:T | C:C | A:A | A:A | C:C | A:A | C:C | G:G | G:G | T:T |
|  | 2011 | *An. coluzzii* | 9 | 277 | C:C | A:A | T:T | A:A | G:G | G:G | T:T | T:C | G:A | T:A | T:C | G:A | T:C | G:G | G:G | T:T |
|  | 2011 | *An. coluzzii* | 1 | 277 | C:C | A:A | T:T | A:A | G:G | G:G | T:T | T:C | G:A | T:T | T:T | G:G | T:T | G:G | G:G | T:T |
|  | 2011 | *An. coluzzii* | 1 | 277 | C:C | A:A | T:T | A:A | G:G | G:G | T:T | T:C | G:G | T:T | T:T | G:G | T:T | G:G | G:G | T:T |
|  | 2011 | *An. coluzzii* | 4 | 277 | C:C | A:A | T:T | A:A | G:G | G:G | T:T | T:T | G:G | T:A | T:C | G:A | T:C | G:G | G:G | T:T |
|  | 2011 | *An. coluzzii* | 15 | 277 | C:C | A:A | T:T | A:A | G:G | G:G | T:T | T:T | G:G | T:T | T:T | G:G | T:T | G:G | G:G | T:T |
|  | 2011 | *An. gambiae* | 1 | 277 | A:A | T:T | C:C | G:G | A:A | T:T | G:G | T:T | G:G | T:T | T:T | G:G | T:T | G:G | G:G | T:T |
|  | 2011 | *An. gambiae* | 4 | 277 | A:A | T:T | C:C | G:G | A:A | T:T | G:G | T:T | G:G | T:T | T:T | G:G | T:T | G:A | G:A | T:C |
|  | 2011 | *An. gambiae* | 1 | 277 | A:A | T:T | C:C | G:G | A:A | T:T | G:G | T:T | G:G | T:A | T:T | G:G | T:T | A:A | A:A | C:C |
|  | 2011 | *An. gambiae* | 239 | 277 | A:A | T:T | C:C | G:G | A:A | T:T | G:G | T:T | G:G | T:T | T:T | G:G | T:T | A:A | A:A | C:C |
|  | 2012 | *An. coluzzii* | 1 | 65 | C:C | A:A | T:T | A:A | G:G | G:G | T:T | T:C | G:A | T:A | T:C | G:A | T:C | G:G | G:G | T:T |
|  | 2012 | *An. gambiae* | 66 | 65 | A:A | T:T | C:C | G:G | A:A | T:T | G:G | T:T | G:G | T:T | T:T | G:G | T:T | A:A | A:A | C:C |
| VK7 | 2006 | *An. coluzzii* | 46 | 178 | C:C | A:A | T:T | A:A | G:G | G:G | T:T | C:C | A:A | A:A | C:C | A:A | C:C | G:G | G:G | T:T |
|  | 2006 | *An. coluzzii* | 1 | 178 | C:C | A:A | T:T | A:A | G:G | G:G | T:T | C:C | A:A | A:A | T:C | G:A | T:C | G:G | G:G | T:T |
|  | 2006 | *An. coluzzii* | 1 | 178 | C:C | A:A | T:T | A:A | G:G | G:G | T:T | C:C | G:A | T:A | T:C | G:A | T:C | G:G | G:G | T:T |
|  | 2006 | *An. coluzzii* | 41 | 178 | C:C | A:A | T:T | A:A | G:G | G:G | T:T | T:C | G:A | T:A | T:C | G:A | T:C | G:G | G:G | T:T |
|  | 2006 | *An. coluzzii* | 1 | 178 | C:C | A:A | T:T | A:A | G:G | G:G | T:T | T:C | G:A | T:A | T:T | G:G | T:T | G:G | G:G | T:T |
|  | 2006 | *An. coluzzii* | 3 | 178 | C:C | A:A | T:T | A:A | G:G | G:G | T:T | T:C | G:G | T:T | T:T | G:G | T:T | G:G | G:G | T:T |
|  | 2006 | *An. coluzzii* | 26 | 178 | C:C | A:A | T:T | A:A | G:G | G:G | T:T | T:T | G:G | T:T | T:T | G:G | T:T | G:G | G:G | T:T |
|  | 2006 | *An. gambiae* | 1 | 178 | A:A | T:T | C:C | G:G | A:A | T:T | G:G | T:T | G:G | T:T | T:T | G:G | T:T | G:A | G:A | T:C |
|  | 2006 | *An. gambiae* | 3 | 178 | A:A | T:T | C:C | G:G | A:A | T:T | G:G | T:T | G:G | T:A | T:T | G:G | T:T | A:A | A:A | C:C |
|  | 2006 | *An. gambiae* | 55 | 178 | A:A | T:T | C:C | G:G | A:A | T:T | G:G | T:T | G:G | T:T | T:T | G:G | T:T | A:A | A:A | C:C |
|  | 2008 | *An. coluzzii* | 11 | 651 | C:C | A:A | T:T | A:A | G:G | G:G | T:T | C:C | A:A | A:A | C:C | A:A | C:C | G:G | G:G | T:T |
|  | 2008 | *An. coluzzii* | 3 | 651 | C:C | A:A | T:T | A:A | G:G | G:G | T:T | C:C | A:A | T:A | T:C | G:A | T:C | G:G | G:G | T:T |
|  | 2008 | *An. coluzzii* | 3 | 651 | C:C | A:A | T:T | A:A | G:G | G:G | T:T | C:C | G:A | T:A | T:C | G:A | T:C | G:G | G:G | T:T |
|  | 2008 | *An. coluzzii* | 3 | 651 | C:C | A:A | T:T | A:A | G:G | G:G | T:T | T:C | G:A | A:A | C:C | A:A | C:C | G:G | G:G | T:T |
|  | 2008 | *An. coluzzii* | 1 | 651 | C:C | A:A | T:T | A:A | G:G | G:G | T:T | T:C | G:A | T:A | T:C | A:A | T:C | G:G | G:G | T:T |
|  | 2008 | *An. coluzzii* | 113 | 651 | C:C | A:A | T:T | A:A | G:G | G:G | T:T | T:C | G:A | T:A | T:C | G:A | T:C | G:G | G:G | T:T |
|  | 2008 | *An. coluzzii* | 14 | 651 | C:C | A:A | T:T | A:A | G:G | G:G | T:T | T:C | G:A | T:T | T:T | G:G | T:T | G:G | G:G | T:T |
|  | 2008 | *An. coluzzii* | 4 | 651 | C:C | A:A | T:T | A:A | G:G | G:G | T:T | T:C | G:G | T:A | T:C | G:A | T:C | G:G | G:G | T:T |
|  | 2008 | *An. coluzzii* | 37 | 651 | C:C | A:A | T:T | A:A | G:G | G:G | T:T | T:C | G:G | T:T | T:T | G:G | T:T | G:G | G:G | T:T |
|  | 2008 | *An. coluzzii* | 6 | 651 | C:C | A:A | T:T | A:A | G:G | G:G | T:T | T:T | G:A | T:A | T:C | G:A | T:C | G:G | G:G | T:T |
|  | 2008 | *An. coluzzii* | 10 | 651 | C:C | A:A | T:T | A:A | G:G | G:G | T:T | T:T | G:G | T:A | T:C | G:A | T:C | G:G | G:G | T:T |
|  | 2008 | *An. coluzzii* | 444 | 651 | C:C | A:A | T:T | A:A | G:G | G:G | T:T | T:T | G:G | T:T | T:T | G:G | T:T | G:G | G:G | T:T |
|  | 2008 | *An. coluzzii* | 1 | 651 | C:C | A:A | T:T | A:A | G:G | G:G | T:T | T:T | G:G | T:T | T:T | G:G | T:T | G:A | G:A | T:C |
|  | 2008 | *An. gambiae* | 1 | 651 | A:A | T:T | C:C | G:G | A:A | T:T | G:G | T:T | G:G | T:T | T:T | G:G | T:T | A:A | A:A | C:C |
|  | 2011 | *An. coluzzii* | 3 | 319 | C:C | A:A | T:T | A:A | G:G | G:G | T:T | C:C | A:A | A:A | C:C | A:A | C:C | G:G | G:G | T:T |
|  | 2011 | *An. coluzzii* | 2 | 319 | C:C | A:A | T:T | A:A | G:G | G:G | T:T | C:C | A:A | T:A | T:C | G:A | T:C | G:G | G:G | T:T |
|  | 2011 | *An. coluzzii* | 3 | 319 | C:C | A:A | T:T | A:A | G:G | G:G | T:T | C:C | G:A | T:A | T:C | G:A | T:C | G:G | G:G | T:T |
|  | 2011 | *An. coluzzii* | 2 | 319 | C:C | A:A | T:T | A:A | G:G | G:G | T:T | C:C | G:G | T:T | T:T | G:G | T:T | G:G | G:G | T:T |
|  | 2011 | *An. coluzzii* | 4 | 319 | C:C | A:A | T:T | A:A | G:G | G:G | T:T | T:C | G:A | A:A | C:C | A:A | C:C | G:G | G:G | T:T |
|  | 2011 | *An. coluzzii* | 42 | 319 | C:C | A:A | T:T | A:A | G:G | G:G | T:T | T:C | G:A | T:A | T:C | G:A | T:C | G:G | G:G | T:T |
|  | 2011 | *An. coluzzii* | 6 | 319 | C:C | A:A | T:T | A:A | G:G | G:G | T:T | T:C | G:A | T:T | T:T | G:G | T:T | G:G | G:G | T:T |
|  | 2011 | *An. coluzzii* | 28 | 319 | C:C | A:A | T:T | A:A | G:G | G:G | T:T | T:C | G:G | T:T | T:T | G:G | T:T | G:G | G:G | T:T |
|  | 2011 | *An. coluzzii* | 4 | 319 | C:C | A:A | T:T | A:A | G:G | G:G | T:T | T:T | G:A | T:A | T:C | G:A | T:C | G:G | G:G | T:T |
|  | 2011 | *An. coluzzii* | 10 | 319 | C:C | A:A | T:T | A:A | G:G | G:G | T:T | T:T | G:G | T:A | T:C | G:A | T:C | G:G | G:G | T:T |
|  | 2011 | *An. coluzzii* | 205 | 319 | C:C | A:A | T:T | A:A | G:G | G:G | T:T | T:T | G:G | T:T | T:T | G:G | T:T | G:G | G:G | T:T |
|  | 2011 | *An. gambiae* | 10 | 319 | A:A | T:T | C:C | G:G | A:A | T:T | G:G | T:T | G:G | T:T | T:T | G:G | T:T | A:A | A:A | C:C |
|  | 2012 | *An. coluzzii* | 3 | 85 | C:C | A:A | T:T | A:A | G:G | G:G | T:T | C:C | A:A | A:A | C:C | A:A | C:C | G:G | G:G | T:T |
|  | 2012 | *An. coluzzii* | 4 | 85 | C:C | A:A | T:T | A:A | G:G | G:G | T:T | C:C | A:A | T:A | T:C | G:A | T:C | G:G | G:G | T:T |
|  | 2012 | *An. coluzzii* | 1 | 85 | C:C | A:A | T:T | A:A | G:G | G:G | T:T | C:C | G:A | T:A | T:C | G:A | T:C | G:G | G:G | T:T |
|  | 2012 | *An. coluzzii* | 1 | 85 | C:C | A:A | T:T | A:A | G:G | G:G | T:T | T:C | G:A | A:A | C:C | A:A | C:C | G:G | G:G | T:T |
|  | 2012 | *An. coluzzii* | 9 | 85 | C:C | A:A | T:T | A:A | G:G | G:G | T:T | T:C | G:A | T:A | T:C | G:A | T:C | G:G | G:G | T:T |
|  | 2012 | *An. coluzzii* | 2 | 85 | C:C | A:A | T:T | A:A | G:G | G:G | T:T | T:C | G:A | T:T | T:T | G:G | T:T | G:G | G:G | T:T |
|  | 2012 | *An. coluzzii* | 2 | 85 | C:C | A:A | T:T | A:A | G:G | G:G | T:T | T:C | G:G | T:T | T:T | G:G | T:T | G:G | G:G | T:T |
|  | 2012 | *An. coluzzii* | 2 | 85 | C:C | A:A | T:T | A:A | G:G | G:G | T:T | T:T | G:G | T:A | T:C | G:A | T:C | G:G | G:G | T:T |
|  | 2012 | *An. coluzzii* | 61 | 85 | C:C | A:A | T:T | A:A | G:G | G:G | T:T | T:T | G:G | T:T | T:T | G:G | T:T | G:G | G:G | T:T |
